# Larger CO_2_ source at the equatorial Pacific during the last deglaciation

**DOI:** 10.1038/srep05261

**Published:** 2014-06-11

**Authors:** Kaoru Kubota, Yusuke Yokoyama, Tsuyoshi Ishikawa, Stephen Obrochta, Atsushi Suzuki

**Affiliations:** 1 Atmosphere and Ocean Research Institute (AORI), the University of Tokyo, 5-1-5 Kashiwa, Chiba 277-8564, Japan; 2 Kochi Institute for Core Sample Research, Japan Agency for Marine-Earth Science and Technology (JAMSTEC), 200 Monobe-otsu, Nankoku, Kochi 783-8502, Japan; 3 Geological Survey of Japan, National Institute of Advanced Industrial Science and Technology (AIST), AIST Tsukuba Central 7, 1-1-1 Higashi, Tsukuba 305-8567, Japan

## Abstract

While biogeochemical and physical processes in the Southern Ocean are thought to be central to atmospheric CO_2_ rise during the last deglaciation, the role of the equatorial Pacific, where the largest CO_2_ source exists at present, remains largely unconstrained. Here we present seawater pH and pCO_2_ variations from fossil *Porites* corals in the mid equatorial Pacific offshore Tahiti based on a newly calibrated boron isotope paleo-pH proxy. Our new data, together with recalibrated existing data, indicate that a significant pCO_2_ increase (pH decrease), accompanied by anomalously large marine ^14^C reservoir ages, occurred following not only the Younger Dryas, but also Heinrich Stadial 1. These findings indicate an expanded zone of equatorial upwelling and resultant CO_2_ emission, which may be derived from higher subsurface dissolved inorganic carbon concentration.

Understanding the past condition of the surface ocean carbonate system and air-sea CO_2_ exchange is crucial to projecting future changes in the carbon cycle under ongoing anthropogenic global warming and ocean acidification. Atmospheric CO_2_ concentration increased by as much as 80 μatm during the last deglaciation, with ~50 μatm released during Heinrich Stadial 1 (HS1, from 17.5 to 14.6 kyr), followed by an additional ~30 μatm during the Younger Dryas (YD, from 12.9 to 11.7 kyr) (ref. 1). While the Southern Ocean is generally considered to be central to the deglacial CO_2_ rise^2^,^3^,^4^,^5^,^6^,^7^,^8^,^9^, the contribution from other oceanic regions remains relatively uninvestigated^10^,^11^,^12^,^13^. Information on the partial pressure of CO_2_ (pCO_2_) is needed to directly constrain past air-sea CO_2_ exchange, and this can be reconstructed from boron isotopes (δ^11^B), a marine carbonate pH proxy^14^,^15^. Regions where surface seawater CO_2_ is out of equilibrium with the atmosphere are ideal for such studies, and the equatorial Pacific is particularly well suited because it represents the largest global CO_2_ source at present (e.g. ref. 16). Corals broadly distributed in tropical to subtropical areas constitute excellent high-resolution geochemical archives for paleo-CO_2_ studies because they may be precisely radiogenically dated (U-series)^3^,^17^, unlike foraminifers that are affected by uncertainty in radiocarbon reservoir age (R).

Two previous studies have attempted to constrain equatorial Pacific CO_2_ changes. Palmer and Pearson^10^ showed increased CO_2_ emission during the last deglaciation in the western equatorial Pacific (WEP) from δ^11^B measurements on the planktonic foraminifer (*Globigerinoides sacculifer*) in a sediment core recovered from offshore Papua New Guinea (ERDC-92, Fig. 1a). Further east, Douville *et al*.^11^ performed δ^11^B on fossil corals from the Marquesas (9.5°S 139.4°W, Fig. 1a) and also demonstrated increased CO_2_ release at the end of the YD. However Douville *et al*.^11^ did not observe a significant CO_2_ release during HS1, complicating interpretation of the equatorial contribution to deglacial atmospheric CO_2_ rise. Integrated Ocean Drilling Program Expedition 310 (IODP Exp. 310)^18^ drilled the outer reef slope at Tahiti (17.6°S 149.5°W, Fig. 1a) recovering fossil corals from an open ocean environment spanning HS1, which enable us to assess the issue.

## Results

### δ^11^B-pH calibration

This study establishes a new empirical δ^11^B-pH calibration utilizing, for the first time, anthropogenic ocean acidification. Empirical calibration is needed to overcome the observed offsets from a theoretical δ^11^B-pH curve in culture experiments for zooxanthellate corals^14^,^15^. (pH is reported using the total hydrogen scale, hereafter pH for simplicity). There are two primary approaches to overcoming the reported offsets. One is an empirical approach^14^ that assumes constant offsets in measured and theoretical δ^11^B (“offset a;” see Methods), while the other is an observational approach^15^ that considers potential pH modification by calcifiers.

The potential for pH-modification is of great concern for δ^11^B-based reconstruction of pH due to the implications for atmospheric pCO_2_ calculation. Such a phenomenon is consistent with indirect pH measurements of internal calcification fluid using pH sensitive dye that suggests a higher pH than ambient seawater, creating better conditions for calcification^19^. A ‘ΔpH' concept in the δ^11^B-pH calibration that reflects pH differences in seawater (pH_SW_) and internal calcification fluid has been proposed^15^, however usage of this proposed relationship to calibrate δ^11^B of modern *Porites* spp. from Tahiti and Marquesas resulted in unrealistically high values (e.g. ~8.34 in AD 1991), well above reported estimates (e.g. refs. 20,21). Therefore the present study employs the empirical equation^14^ here (see Methods).

Ocean acidification was estimated from a combination of *in situ* fCO_2_ values in the surface ocean, atmospheric CO_2_ concentration directly measured at the Mauna Loa observatory in Hawaii since AD 1960 (ref. 22), and CO_2_ concentrations within bubbles trapped in an Antarctic ice core^23^ (Figs. 2, S1–S4; Supplementary Methods). These data were then fit to the previously reported δ^11^B measurements of *Porites* spp. (refs. 11,24), which are for the years AD 1991, 1950, and 1700 (Fig. 2, see Methods and Supplementary Methods for details of the δ^11^B-pH calibration and pH estimation since the Industrial Revolution).

### pH and pCO_2_ reconstruction

Using our revised calibration, we reconstructed pH from our new δ^11^B measurements on Tahitian corals, as well as from previously reported data^11^ from both the Marquesas and Tahiti, and the overall result is consistent with the WEP foraminifer δ^11^B variations^10^ (Fig 3a and b). The oldest coral sample, dated to 20.7 ka BP during the last glacial maximum (LGM), exhibits a relatively high pH (8.26). From 15.5 to 9.0 ka BP, pH is generally constant within uncertainty (8.15–8.22) and consistent with the preindustrial value of 8.20. Four notable pH excursions are associated with HS1 and the YD. Two of our samples exhibit anomalously low pH at the end of HS1 (8.13 at 15.15 ka and 8.09 at 14.99 ka BP), in addition to those at end of the YD at the Marquesas^11^. The low pH following HS1 had been previously undetected at this location. Calculation of pCO_2_ (see Methods) reveals deglacial values significantly above those of the atmosphere (Figs. 3c and 4a). Conversely, ΔpCO_2_ during last glacial and the early Holocene was nearly zero, suggesting air-sea CO_2_ equilibrium.

**Figure 1:**
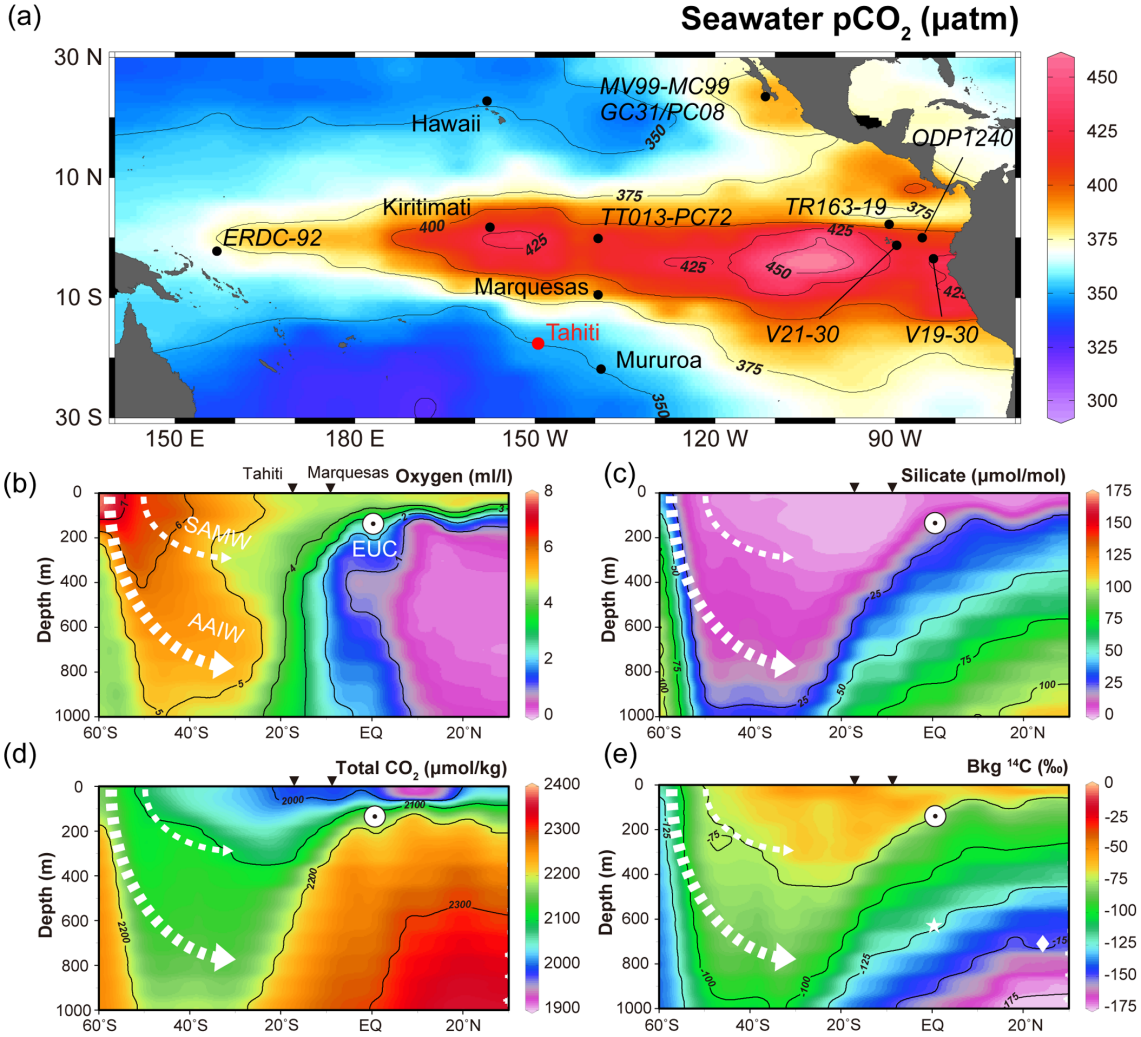
Sea surface pCO_2_ in the equatorial Pacific and vertical sections of the South Pacific. (a) Equatorial Pacific locations discussed in the text with sea surface pCO_2_ for the reference year AD 2000 in which red and blue represent CO_2_ sources and sinks, respectively^16^. Vertical sections from 60°S to 30°N of (b) oxygen, (c) silicate, (d) DIC, and (e) Δ^14^C. All data are downloaded and plotted using Ocean Data View software, version 4.5.5 (ref. 60). Inverted black triangles show locations of Tahiti and Marquesas. The white star and diamond in **e** indicate the locations of cores MV99-MC99/GC31/PC08 (refs. 36,43) and V21–31 (ref. 42), respectively.

Results from a different portion of the same 14.99 ka BP coral sample deviate by as much as 1.4‰, which corresponds to 0.11 in pH and 100 μatm in pCO_2_ (310-M0024A-11R-1W_77-90 and 310-M0024A-11R-1W_60-75, Table S1). Considering the average ~4 year temporal resolution of each sample, these excursions occurred abruptly and persisted for several years, which differs from modern observations that show no clear interannual or decadal variability (Supplementary Methods, Fig. S1). This enhanced variability, which is also observed in Sr/Ca derived SST results from another *Porites* colony recovered from IODP Exp. 310 (ref. 25), may relate to Tahiti's location at the rim of equatorial upwelling cell (Fig. 1a). Taken together, pCO_2_ (pH) records indicate that the equatorial Pacific became a larger CO_2_ source during the last deglaciation with excursions at the end of HS1 and the YD.

### Marine ^14^C reservoir age compilation

Compiled marine ^14^C reservoir ages (R) throughout the equatorial Pacific resemble pCO_2_ variability (see Methods; Figs. 4 and S5; Table S2). Larger and more variable values of R are evident in Tahiti during HS1 and the YD, and enhanced R variability is also seen in the Marquesas (Figs. 4 and S5). Paterne *et al*.^26^ sub-sampled different parts in the same fossil coral skeleton and analyzed both ^14^C and U/Th. They observed no difference in U/Th dates, but a much larger difference in ^14^C. Possibilities of either a diagenetic alteration or a change in R were suggested. The latter is more probable because a large variation in R is also suggested from Vanuatu coral at 11.7–12.4 ka (~400 years; during the YD, ref. 27).

Though reported R_diff_ (difference between calculated R and modern R; see Methods) data around the upwelling zone during the LGM are sparse, calculations with the new Lake Suigetsu datasets^28^ suggests no substantial change in R (see Methods; Figs. 4 and S5). This implies that the CO_2_ exchange rate in the surface equatorial Pacific during the last glacial was almost the same as present, which supports the above-mentioned observation that ΔpCO_2_ is essentially equivalent to zero and indicates that anomalous R values are limited to the last deglaciation.

## Discussion

pCO_2_ variability in subtropical oligotrophic water can be explained by mixing of water masses that exhibit distinctly different dissolved inorganic carbon (DIC) concentrations. A southward migration of the intertropical convergence zone (ITCZ) that partly controls thermocline depth is hypothesized during Heinrich Events including HS1 and the YD (e.g. ref. 29). At present, the ITCZ does not seem to affect surface pCO_2_ variability (Fig. 1a), and if it is displaced southward, the locus of equatorial upwelling remains at the equator due to the influence of inter-hemispheric asymmetry of Coriolis force (e.g. refs. 30,31). Enhanced upwelling (shallower thermocline, La Niña-like conditions) or increased subsurface DIC concentration are more likely to drive pCO_2_ variability based on sedimentary evidence from the equatorial Pacific for higher nutrient content, e.g., enhanced biogenic opal export production and lower stable carbon isotopes (δ^13^C) (TT013-PC72, ODP Site 1240 and TR163-19)^32^,^33^,^34^,^35^ (Fig. 1a). Semi-conservative radiogenic neodymium isotopes (εNd) from sediment cores at the eastern equatorial Pacific (EEP) (ODP Site 1240) and off Baja California (MV99-MC99-GC31/PC08) indicate stronger subsurface water transport from the south^33^,^36^ (Fig. 1). Covariation of geochemical properties between the Southern Ocean and the equatorial Pacific suggest a subsurface connection during the last deglaciation (e.g. refs. 32,33,34,35,36,37,38). Thus, pCO_2_ variability may be explained by an increase in DIC in the upwelled, subsurface water masses as opposed to physical processes alone.

Water mass subduction along the subantarctic front, mainly off Chile^39^, forms Subantarctic Mode Water (SAMW) and Antarctic Intermediate Water (AAIW) that upwells at the equatorial Pacific via the Equatorial Undercurrent (EUC) (Fig. 1). SAMW and AAIW are characterized by higher/lower concentrations of oxygen/silicic acid (Fig. 1b,c). It is suggested that the abyssal DIC reservoir around the Southern Ocean increased during the last glacial period^6^,^7^,^8^,^40^, which would have contributed to lower atmospheric pCO_2_. Carbon dioxide was released to the surface through deep ocean ventilation during HS1 and the YD (refs. 2,3,4,5,6,7,8,9), however export production was insufficient to fully compensate the increased carbon flux^41^. This is consistent with residual radiocarbon content (Δ^14^C) of intermediate water at the EEP (V21–30)^42^ and off Baja California (MV99-MC99-GC31/PC08)^43^ that indicates anomalously older water was incorporated into SAMW/AAIW (Fig. 1), as well as with depleted δ^13^C of surface and lower thermocline dwelling foraminifers from sediment cores at both equatorial (TT013-PC72, ODP Site 1240 and TR163-19) and South Pacific sites^32^,^33^,^34^,^35^,^37^,^38^. Moreover, enhanced export production of biogenic opal suggest more silicic acid was transported via the EUC to thermocline water at the equatorial Pacific (V19-30 and TT013-PC72) without being consumed completely within the Southern Ocean^4^,^35^,^41^. Stronger Ekman transport in association with sea ice retreat and a poleward shift of southern westeries is suggested to be a driver^4^,^5^.

A similarity between R and pCO_2_ variability during the last deglaciation supports an interpretation that older DIC was incorporated to subtropical surface water through mixing with SAMW/AAIW, though, contrary evidence comes from the current formation sites off Chile^44^ and New Zealand^6^. However, a key sediment record off Chile was recently reevaluated, and the new interpretations indicate stronger upwelling and subsequent larger R in surface water in the Southern Ocean^9^, which agrees well with our interpretation. Yet, further work is still needed to fully understand both the physical and biogeochemical dynamics in the Southern Ocean and the equatorial Pacific^2^.

Positive ΔpCO_2_ indicates CO_2_ flux from the ocean to the atmosphere. Previous studies^10^,^11^ indicated that the equatorial Pacific contributed to deglacial CO_2_ rise, however the timing of anomalously higher pCO_2_ events recorded in radiogenically dated fossil corals do not systematically correspond to those of atmospheric CO_2_ rise recovered from Antarctic ice core on the GICC05 timescale^1^ (Figs. 3 and 4). Moreover our new calibration reveals a modest CO_2_ emission continued through the Bølling/Allerød/Antarctic Cold Reversal when no atmospheric CO_2_ increase is observed (Figs. 3 and 4). Though we demonstrate that the equatorial Pacific became a larger CO_2_ source during the last deglaciation, it is too early to conclude its exact contribution to atmospheric CO_2_ rise. The Southern Ocean is suggested to be central in CO_2_ degassing^4^,^5^,^6^,^7^,^8^,^9^ and the contribution of the terrestrial biosphere should be further evaluated^45^. More evidence spanning the YD and the early part of HS1, in particular the sharp rise in atmospheric CO_2_ and the sudden drop of δ^13^C of CO_2_ (refs. 1,46), as well as more spatial coverage is needed.

## Methods

### δ^11^B analyses

The δ^11^B values of fossil *Porites* spp. were measured following the protocol of Ishikawa and Nagaishi^47^. Fossil corals were screened for diagenetic alteration with X-ray diffraction and geochemical analyses, as well as visual using a Scanning Electron Microscope^48^. Bulk sampling was conducted along the growth axis, and time resolution of each sample is several years (1–8 years) depending on growth rate of each coral^48^. Typically 6 mg of carbonate was used for δ^11^B measurement. After removals of organic matter using 30% H_2_O_2_ for ~12 hours, boron was purified by cation and anion exchange using AG 50 W X12 and 1-X4 resin (Bio-Rad, USA) and then δ^11^B were measured using the positive polarity thermal ionization mass spectrometer (P-TIMS; Thermo Finnigan *TRITON*) installed at Kochi Core Center, Japan Agency for Marine-Earth Science and Technology. All reported δ^11^B values are the mean of duplicate analyses (Table S1). Repeated analysis of the JCp-1, carbonate standard provided by Geological Survey of Japan yielded 24.21 ± 0.18‰ (2σ, n = 18), which is the finest precision to date^47^. Differences between the duplicates are 0.08‰ on average with the largest one of 0.28‰ (Table S1), which is within the measurement uncertainty of JCp-1. We conservatively report ±0.18‰ as the analytical uncertainty of our δ^11^B measurements.

### δ^11^B-pH calibration and pCO_2_ calculation

First, the analytical procedure-specific isotopic offset^49^ was corrected using the equation bellow (modified after Zeebe & Wolf-Gladrow^50^) in order to reduce inter-laboratory offsets in reported δ^11^B values, 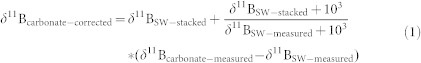
where δ^11^B_carbonate-corrected_ is the boron isotopic value of carbonate after correction and δ^11^B_SW-stacked_ is the global average δ^11^B of seawater. We used the recommended value of 39.61‰ for δ^11^B_SW-stacked_ (ref. 49). δ^11^B_carbonate-measured_ and δ^11^B_SW-measured_ are δ^11^B of carbonate samples and of seawater measured in different laboratories. Without performing inter-laboratory correction of JCp-1, results of Douville *et al*.^11^ and this study differ by 0.25‰, equivalent to ~0.03 pH units. After the correction using the reported value of δ^11^B_SW-measured_ (refs. 11,47), the difference improved to be 0.07‰, indicating that correction should be performed.

**Figure 2:**
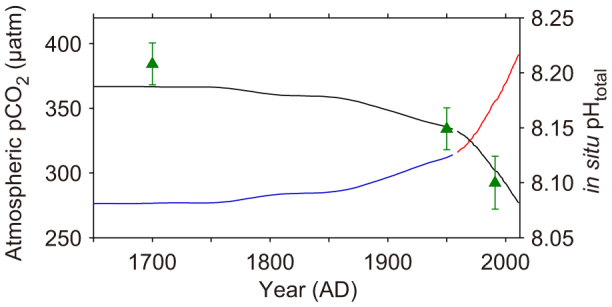
Measured pCO_2_ from Mauna Loa^22^ and Law Dome ice core^23^ (red and blue lines) and estimated pH variation at Tahiti (black line). Green triangles are newly calibrated pH from previously reported δ^11^B of modern corals from Tahiti and Marquesas^11^,^24^ (Table S1). Estimated pH from the AD 1700 Marquesas coral was scaled by 0.04 to correct for offset from Tahitian coral values. Error bars are 2σ.

**Figure 3:**
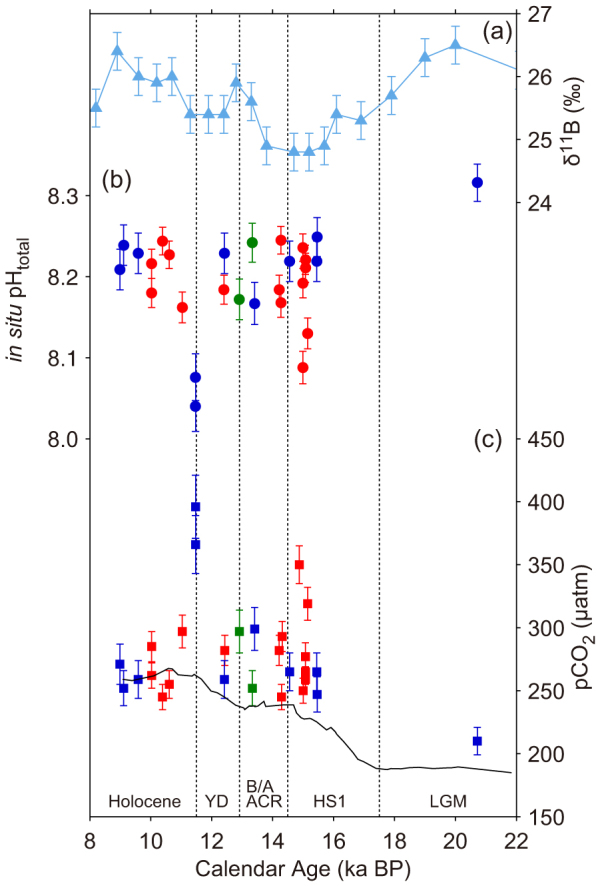
Deglacial δ^11^B, pH and pCO_2_ variability in the equatorial Pacific. (a) Reported δ^11^B values of planktonic foraminifera with 2σ uncertainty from ERDC-92 (ref. 10). Age model is from the original publication. (b) *In situ* pH reconstructed from δ^11^B of fossil *Porites* spp. using our new calibration. Red and green circles are from Tahiti by this study and Douville *et al*.^11^, respectively. Blue circles are from Marquesas by Douville *et al*.^11^ after correction by +0.04 pH units. (c) Derived pCO_2_ of surface water around equatorial South Pacific Ocean (same colors as b) and atmospheric pCO_2_ on the GICC05 timescale^1^. All error bars are 2σ. YD, Younger Dryas; B/A, Bølling/Allerød; ACR, Antarctic Cold Reversal; HS1, Heinrich Stadial 1; LGM, Last Glacial Maximum.

After the correction, the empirical calibration equation reported by Hönisch *et al*.^14^ was utilized. In this equation, the vital offset ‘a' and fractionation factor ‘α_3-4_' that yield the lowest e_rms_ (root-mean square error) is determined. 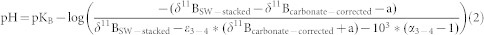
Three previously reported δ^11^B values for modern *Porites* spp. from Tahiti and Marquesas were fit to estimated pH at AD 1991, 1950 and 1700 (refs. 11,24) (Fig. 2, Table S1). In the calibration ‘a' was determined as ‘−6.0' when we chose recently reevaluated α_3-4_ of ‘1.0272' (ref. 51). When this calibration was conducted, both ‘a' and ‘α_3-4_' were allowed to vary freely, and the resultant α_3-4_ with the lowest e_rms_ was very close to that of Klochko *et al*.^51^ rather than the previously accepted value of Kakihana *et al*.^52^ Thus we used Klochko's fractionation factor. pH was calculated from both previously reported and newly obtained δ^11^B values considering a local difference of seawater pH between Tahiti and Marquesas (see Supplementary Methods for details).

pCO_2_ was further calculated from obtained pH values from δ^11^B_carbonate-corrected_ using CO2SYS program under assumptions of constant temperature, salinity and total alkalinity (see also Supplementary Methods for details). The δ^11^B values of *G. sacclifer*^10^ are not included here due to large uncertainties in the δ^11^B-pH calibration (e.g. refs. 12,15).

### R compilation

**Figure 4:**
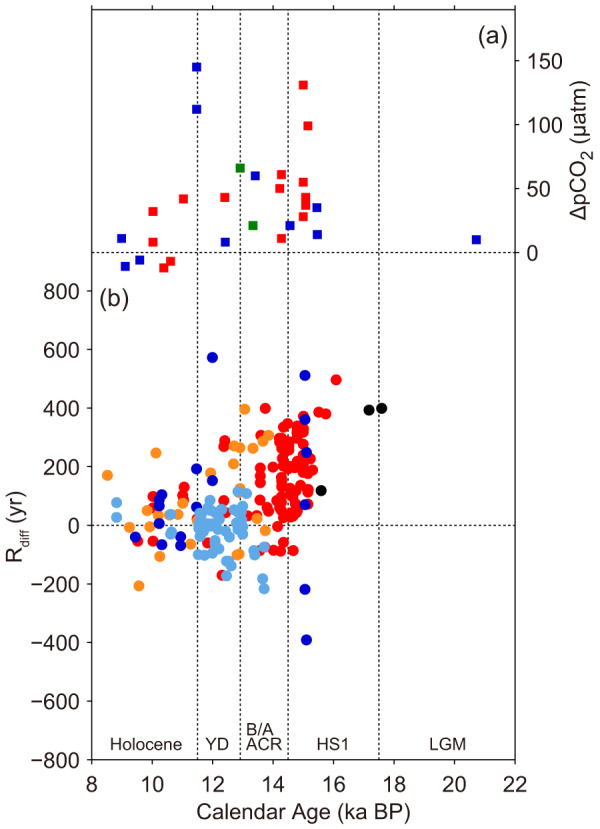
Differences between modern and deglacial pCO_2_ and R (ΔpCO_2_ and R_diff_). The last deglaciation is characterized by larger and more variable pCO_2_ and R values. (a) Atmosphere-ocean ΔpCO_2_ based on Tahiti and Marquesas corals and Antarctic ice core^1^. Figure legend is same as Fig. 3. (b) R_diff_ around the equatorial Pacific Ocean calculated from fossil corals (red: offshore Tahiti; orange: reef crest of Tahiti barrier reef^58^, blue: Marquesas^26^; light blue: Kiritimati^59^; black: Mururoa^58^). Horizontal dashed lines represent ΔpCO_2_ = 0 and R_diff_ = 0.

Published ^14^C (radiocarbon years) and U/Th ages of fossil coral samples obtained during IODP Exp. 310 were compiled in order to calculate residual radiocarbon activities (Δ^14^C) and R. We verified via IODP sample ID and core photographs^18^ that the exact same samples were selected (Table S2). In some cases different portion of the skeleton of the same coral was dated. Given that lifetimes of coral are generally less than several decades, temporal gaps derived from sub-sampling are negligible in calculations of Δ^14^C and R. We did not use ^14^C ages from either microbialite (carbonate created by bacteria) or encrusting coralline algae from equivalent down-core depths due to a possibility of post-depositional growth (for details, see ref. 53). Calculation was done according to equations (3) and (4) where ^14^C-age is an original radiocarbon data before a local R correction^54^,^55^. 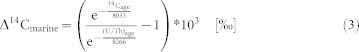

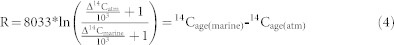
Atmospheric Δ^14^C (Δ^14^C_atm_) from INTCAL09 (ref. 56) was used to calculate R. Samples that span 29–30 ka BP were calculated using the recently published Lake Suigetsu Δ^14^C_atm_ dataset^28^, since as much as ~100‰ offsets are observed between INTCAL09 in this interval (Fig. S5a). Southon *et al*.^57^ points out that the INTCAL09 curve heavily relies on Cariaco Basin varve sediment data beyond the dendro-chronological limit (>12.55 ka BP) and has deficits during the YD and HS1 due to changes in local R in the equatorial Atlantic. Specifically, a difference during 15.5–17.0 ka BP is apparent, however it does not affect our calculation since there are no samples that span this interval (Fig. S5). Other ^14^C and U/Th datasets for fossil corals from the equatorial Pacific islands were also used to calculate R including Tahiti^58^, Marquesas^26^, Kiritimati^59^ and Mururoa^58^ (Figs. 1, 4, and S5b). R_diff_ denotes differences between calculated R and modern R (235 ± 110 for Tahiti^56^, 390 ± 60 for Marquesas^26^, 335 ± 100 for Kiritimati^56^, 300 ± 100 for Mururoa^58^), thus it differs from ΔR that conventionally represents local ^14^C reservoir age. We estimate that the accumulated uncertainty in the R_diff_ calculation are the sum of errors in ^14^C dating, U/Th dating, and modern R (Fig. S5b; Table S2).

## Author Contributions

Y.Y. designed the study and obtained samples. K.K. and T.I. measured coral boron isotope ratios. A.S. prepared samples. S.O. performed the statistical analysis of the data. K.K., Y.Y., T.I., S.O. and A.S. contributed to the interpretation and the preparation of the final manuscript.

## Supplementary Material

Supplementary Information Supplementary Information

## References

[b1] Lourantou A. *et al.* Constraint of the CO_2_ rise by new atmospheric carbon isotopic measurements during the last deglaciation. Glob. Biogeochem. Cycles 24, GB003545 (2010).

[b2] Matsumoto K. & Yokoyama Y. Atmospheric Δ^14^C reduction in simulations of Atlantic overturning circulation shutdown. Glob. Biogeochem. Cycles 27, 296–304 (2013).

[b3] Yokoyama Y. & Esat T. M. Global climate and sea level- enduring variability and rapid fluctuations over the past 150,000 years. Oceanography 24, 54–69 (2011).

[b4] Anderson R. F. *et al.* Wind-Driven Upwelling in the Southern Ocean and the Deglacial Rise in Atmospheric CO_2_. Science 323, 1443–1448 (2009). 19286547 10.1126/science.1167441

[b5] Toggweiler J. R., Russel J. L. & Carson S. R. Midlatitude westerlies, atmospheric CO_2_, and climate change during the ice ages. Paleoceanography 21, PA2005 (2006).

[b6] Rose K. A. *et al.* Upper-ocean-to-atmosphere radiocarbon offsets imply fast deglacial carbon dioxide release. Nature 466, 1093–1097 (2010). 20740012 10.1038/nature09288

[b7] Burke A. & Robinson L. F. The Southern Ocean's Role in Carbon Exchange During the Last Deglaciation. Science 335, 557–561 (2012). 22174131 10.1126/science.1208163

[b8] Skinner L. C., Fallon S., Waelbroeck C., Michel E. & Barker S. Ventilation of the Deep Southern Ocean and Deglacial CO_2_ Rise. Science 328, 1147–1151 (2010). 20508128 10.1126/science.1183627

[b9] Siani G. *et al.* Carbon isotope records reveal precise timing of enhanced Southern Ocean upwelling during the last deglaciation. Nat. Commun. 4:2758, 10.1038/ncomms3758 (2013). 10.1038/ncomms3758 24202198

[b10] Palmer M. R. & Pearson P. N. A 23,000-Year Record of Surface Water pH and pCO_2_ in the Western Equatorial Pacific Ocean. Science 300, 480–482 (2003). 12663815 10.1126/science.1080796

[b11] Douville E. *et al.* Abrupt sea surface pH change at the end of the Younger Dryas in the central sub-equatorial Pacific inferred from boron isotope abundance in corals (*Porites*). Biogeosciences 7, 2445–2459 (2010).

[b12] Henehan M. J. *et al.* Calibration of the boron isotope proxy in the planktonic foraminifera *Globigerinoides ruber* for use in palaeo-CO_2_ reconstruction. Earth Planet. Sci. Lett. 364, 111–122 (2013).

[b13] Yu J., Thornalley D. J. R., Rae J. W. B. & McCave N. I. Calibration and application of B/Ca, Cd/Ca, and δ^11^B in *Neogloboquadrina pachyderma* (sinistral) to constrain CO_2_ uptake in the subpolar North Atlantic during the last deglaciation. Paleoceanography 28, 237–252 (2013).

[b14] Hönisch B., Hemming N. G. & Loose B. Comment on ‘A critical evaluation of the boron isotope-pH proxy: the accuracy of ancient ocean pH estimates' by M. Pagani, D. Lemarchand, A. Spivak, J. Gaillardet. Geochim. Cosmochim. Acta 71, 1636–1641 (2007).

[b15] Trotter J. *et al.* Quantifying the pH ‘vital effect' in the temperate zooxanthellate coral *Cladocora caespitosa*: Validation of the boron seawater pH proxy. Earth Planet. Sci. Lett. 303, 163–173 (2011).

[b16] Takahashi T. *et al.* Climatological mean and decadal change in surface ocean pCO_2_, and net sea–air CO_2_ flux over the global oceans. Deep-Sea Res. II 56, 554–577 (2009).

[b17] Deschamps P. *et al.* Ice-sheet collapse and sea-level rise at the Bølling warming 14,600 years ago. Nature 483, 559–564 (2012). 22460900 10.1038/nature10902

[b18] Camoin G. F., Iryu Y., McInroy D. & the IODP Expedition 310 Scientists. IODP Expedition 310 Reconstructs Sea Level, Climatic, and Environmental Changes in the South Pacific during the Last Deglaciation. Sci. Drill. 5, 4–12 (2007).

[b19] Venn A. A. *et al.* Impact of seawater acidification on pH at the tissue–skeleton interface and calcification in reef corals. Proc. Natl. Acad. Sci. USA 110, 1634–1639 (2013). 23277567 10.1073/pnas.1216153110 PMC3562847

[b20] Caldeira K. & Wickett M. E. Anthropogenic carbon and ocean pH. Nature 425, 365 (2003). 14508477 10.1038/425365a

[b21] Key R. M. *et al.* A global ocean carbon climatology: Results from Global Data Analysis Project (GLODAP). Glob. Biogeochem. Cycle 18, GB002247 (2004).

[b22] Keeling R. F., Piper S. C., Bollenbacher A. F. & Walker J. S. Atmospheric CO_2_ records from sites in the SIO air sampling network. In Trends: A Compendium of Data on Global Change. Carbon Dioxide Information Analysis Center, Oak Ridge National Laboratory, U.S. Department of Energy, Oak Ridge, Tenn., U.S.A. 10.3334/CDIAC/atg.035 (2009).

[b23] Etheridge D. M. *et al.* Natural and anthropogenic changes in atmospheric CO_2_ over the last 1000 years from air in Antarctic ice and firn. J. Geophys. Res. 101, 4115–4128 (1996).

[b24] Gaillardet J. & Allègre C. J. Boron isotopic compositions of corals: seawater or diagenesis record? Earth Planet. Sci. Lett. 136, 665–676 (1995).

[b25] Felis T. *et al.* Pronounced interannual variability in tropical South Pacific temperatures during Heinrich Stadial 1. Nat. Commun. 3:965, 10.1038/ncomms1973 (2012). 10.1038/ncomms1973 22828625

[b26] Paterne M. *et al.* Paired ^14^C and ^230^Th/U dating of surface corals from the Marquesas and Vanuatu (sub-equatorial Pacific) in the 3000 to 15,000 cal yr interval. Radiocarbon 46, 551–566 (2004).

[b27] Burr G. S. *et al.* A high-resolution radiocarbon calibration between 11,700 and 12,400 calendar years BP derived from ^230^Th ages of corals from Espiritu Santo island, Vanuatu. Radiocarbon 40, 1093–1105 (1998).

[b28] Bronk-Ramsey C. *et al.* A Complete Terrestrial Radiocarbon Record for 11.2 to 52.8 kyr B.P. Science 338, 370–374 (2012). 23087245 10.1126/science.1226660

[b29] Deplazes G. *et al.* Links between tropical rainfall and North Atlantic climate during the last glacial period. Nature Geosci. 6, 213–217 (2013).

[b30] Talley L. D., Pickard G. L., Emery W. J. & Swift J. H. (2011) in Descriptive Physical Oceanography: An Introduction.(Elsevier Academic Press, Amsterdam).

[b31] Sarmiento J. L. & Gruber N. in Ocean Biogeochemical Dynamics. (Prinston Univ. Press, 2011).

[b32] Spero H. J. & Lea D. W. The Cause of Carbon Isotope Minimum Events on Glacial Terminations. Science 296, 522–525 (2002). 11964477 10.1126/science.1069401

[b33] Pena L. D. *et al.* Rapid changes in meridional advection of Southern Ocean intermediate waters to the tropical Pacific during the last 30 kyr. Earth Planet. Sci. Lett. 368, 20–32 (2013).

[b34] Pena L. D., Cacho I., Ferretti P. & Hall M. A. El Nino–Southern Oscillation–like variability during glacial terminations and interlatitudinal teleconnections. Paleoceanography 23, PA001620 (2008).

[b35] Hayes C. T., Anderson R. F. & Fleisher M. Q. Opal accumulation rates in the equatorial Pacific and mechanisms of deglaciation. Paleoceanography 26, PA002008 (2011).

[b36] Basak C., Martin E. E., Horikawa K. & Marchitto T. M. Southern Ocean source of ^14^C-depleted carbon in the North Pacific Ocean during the last deglaciation. Nature Geosci. 3, 770–773 (2010).

[b37] Loubere P. & Bennet S. Southern Ocean biogeochemical impact on the tropical ocean: Stable isotope records from the Pacific for the past 25,000 years. Glob. Planet. Change 63, 333–340 (2008).

[b38] Bostock H. C., Opdyke B. N., Gagan M. K. & Fifield L. K. Carbon isotope evidence for changes in Antarctic Intermediate Water circulation and ocean ventilation in the southwest Pacific during the last deglaciation. Paleoceanography 19, PA001047 (2004).

[b39] Bostock H. C., Sutton P. J., Williams M. J. M. & Opdyke B. N. Reviewing the circulation and mixing of Antarctic Intermediate Water in the South Pacific using evidence from geochemical tracers and Argo float trajectories. Deep-Sea Res. I 73, 84–98 (2013).

[b40] Sikes E. L., Samson C. R., Guilderson T. P. & Howard W. R. Old radiocarbon ages in the southwest Pacific Ocean during the last glacial period and deglaciation. Nature 405, 555–559 (2000). 10850711 10.1038/35014581

[b41] Horn M. G., Beucher C. P., Robinson R. S. & Brezeyinski M. A. Southern ocean nitrogen and silicon dynamics during the last deglaciation. Earth Planet. Sci. Lett. 310, 334–339 (2011).

[b42] Stott L., Southon J., Timmermann A. & Koutavas A. Radiocarbon age anomaly at intermediate water depth in the Pacific Ocean during the last deglaciation. Paleoceanography 24, PA001690 (2009).

[b43] Marchitto T. M., Lehman S. J., Ortiz J. D., Flückiger J. & van Green A. Marine Radiocarbon Evidence for the Mechanism of Deglacial Atmospheric CO_2_ Rise. Science 316, 1456–1459 (2007). 17495139 10.1126/science.1138679

[b44] De Pol-Holz R., Keigwin L., Southon J., Hebbelin D. & Mohtadi M. No signature of abyssal carbon in intermediate waters off Chile during deglaciation. Nature Geosci. 3, 192–195 (2010).

[b45] Ciais P. *et al.* (2012) Large inert carbon pool in the terrestrial biosphere during the Last Glacial Maximum. Nature Geosci 5, 74–79.

[b46] Schmitt J. *et al.* Carbon Isotope Constraints on the Deglacial CO_2_ Rise from Ice Cores. Science 336, 711–714 (2012). 22461496 10.1126/science.1217161

[b47] Ishikawa T. & Nagaishi K. High-precision isotopic analysis of boron by positive thermal ionization mass spectrometry with sample preheating. J. Anal. At. Spectrom. 26, 359–365 (2011).

[b48] Inoue M. *et al.* Trace element variations in fossil corals from Tahiti collected by IODP Expedition 310: Reconstruction of marine environments during the last deglaciation (15 to 9 ka). Mar. Geol. 271, 303–306 (2010).

[b49] Foster G. L., Pogge von Strandmann P. A. E. & Rae J. W. B. Boron and magnesium isotopic composition of seawater. Geochem. Geophys. Geosyst. 11, GC003201 (2010).

[b50] Zeebe R. E. & Wolf-Gladrow D. in CO_2_ in Seawater: Equilibrium, kinetics, isotopes. (Elsevier Oceanography Series, 2001).

[b51] Klochko K. *et al.* Experimental measurement of boron isotope fractionation in seawater. Earth Planet. Sci. Lett. 248, 276–285 (2006).

[b52] Kakihana H., Kotaka M., Satoh S., Nomura M. & Okamoto M. Fundamental Studies on the Ion-Exchange Separation of Boron Isotopes. Bull. Chem. Soc. Japan 50, 158–163 (1977).

[b53] Seard C. *et al.* Microbialite development patterns in the last deglacial reefs from Tahiti (French Polynesia; IODP Expedition #310): Implications on reef framework architecture. Mar. Geol. 279, 63–86 (2011).

[b54] Adkins J. F. & Boyle E. A. Changing atmospheric Δ^14^C and the record of deepwater paleoventilation ages. Paleoceanography 12, 337–344 (1997).

[b55] Burr G. S. *et al.* Modern and Pleistocene reservoir ages inferred from South Pacific Corals. Radiocarbon 51, 319–335 (2009).

[b56] Reimer P. J. *et al.* IntCal09 and Marine09 radiocarbon age calibration curves, 0–50,000 years cal BP. Radiocarbon 51, 1111–1150 (2009).

[b57] Southon J., Noronha A. L., Cheng H., Edwards R. L. & Wang Y. A high-resolution record of atmospheric ^14^C based on Hulu Cave speleothem H82. Quat. Sci. Rev. 33, 32–41 (2012).

[b58] Bard E., Arnold M., Hamelin B., Tisnerat L. N. & Cabioch G. Radiocarbon calibration by means of mass spectrometric ^230^Th/^234^U and ^14^C ages of corals: an updated database including samples from Barbados, Mururoa and Tahiti. Radiocarbon 40, 1085–1092 (1998).

[b59] Fairbanks R. G. *et al.* Radiocarbon calibration curve spanning 0 to 50,000 years BP based on paired ^230^Th/^234^U/^238^U and ^14^C dates on pristine corals. Quat. Sci. Rev. 24, 1781–1796 (2005).

[b60] Schlitzer R. Ocean Data View. <http://odv.awi.de>, (2014) Date of access:29/01/2014.

