# Supplementary material for: Larger CO2 source at the equatorial Pacific during the last deglaciation
Source: Sci Rep. 2014 Jun 11;4:5261. doi: 10.1038/srep05261 (PMC4052749; doi:10.1038/srep05261)
Supplement: Supplementary Information [file srep05261-s1.doc]

**Supplementary Information**

**Larger CO_2_ source at the equatorial Pacific during the last deglaciation**

Kaoru Kubota, Yusuke Yokoyama, Tsuyoshi Ishikawa, Stephen Obrochta, Atsushi Suzuki

**Supplementary Methods**

**Supplementary Figures S1–S9**

**Supplementary Tables S1–S2**

**Supplementary References**

**Supplementary Methods**

**Estimation of seasonal pH variations around Tahiti, Hawaii and Marquesas and ocean acidification after the Industrial Revolution.**

Seasonal pH variations around Tahiti (17.6ºS 149.5ºW), Hawaii (22.75ºN 158.0ºW) and Marquesas (9.5ºS 139.4ºW) were estimated using total alkalinity (TA) and fugacity of CO_2_ (fCO_2_) as provided by the Surface Ocean CO_2_ Atlas^61^ because monthly or annually continuous observations are limited to specific locations such as Hawaii^62^ and Bermuda^63^. We extracted data from 2.5º latitude by 5.0º longitude grids centered on each island. Because chemical properties of surface seawater are relatively meridionally homogeneous in the subtropical Pacific Ocean, the enlarged longitudinal range allowed extraction of as much data as possible. We calculated TA from an empirical equation for global (sub) tropics obtained from high quality seawater carbonate chemistry datasets from AD 1990s (ref. 64). The equation is as bellow.

$TA=2305+58.66*(SSS-35)+2.32*(SSS-35)^{2}-1.41*(SST-20)+0.040*(SST-20)^{2}$(S1)

Where SST and SSS are Sea surface temperature and salinity, respectively. We used directly measured SST and SSS data from each SOCAT cruise. For grid points with no data or unreasonably extreme salinity values, we imported salinity values from the closest grid point in the SODA dataset^65^.

We calculated other CO_2_ parameters using the CO2SYS program, version 1.0 (ref. 66) using the dissociation constants for carbonic acid (CO_3_^2-^, HCO_3_^-^) of Lueker *et al*.^67^ and for hydrogen sulfate (HSO_4_^-^) of Dickson^68^. The total hydrogen pH scale is used^50,66^ (hereafter ‘pH’ for simplicity). The same calculation was also performed using DIC, SST and SSS from the Hawaii Ocean Time–Series^62,69^, which has been continuously measured at Station ALOHA (22˚ 45'N, 158˚ 00'W) since AD 1990. In this calculation, TA was estimated with equation (S1) using SST and SSS measured at Station ALOHA.

DIC calculated from the above calculations are salinity-normalized (nDIC) because DIC is influenced by condensation/dilution of seawater^62,70^. nDIC was obtained by multiple regression analysis following Ishii *et al*.^70^ The calculated nDIC was fitted as empirical functions of a timing of observation (yr) and physical parameters of SST and SSS:

$$\begin{matrix} nDIC=DIC*35/SSS \\ =f (yr,SST,SSS) \\ =C_{0}+C_{1}*yr+C_{2}*temp+C_{3}*temp^{2}+C_{4}*temp^{3}+C_{5}*sal+\varepsilon\end{matrix}$$

(S2)

Where *yr* = year - 1991.5, *temp* = SST - T_ave_, and *sal* = SSS - 35. For SST, average temperature (T_ave_) was separately specified for Tahiti, Hawaii and Marquesas. The terms *C_0_* ~ *C_5_* are coefficients of multiple regressions, and *ε* represents the residual of the fitting. The polynomial of *temp* in the equation exhibits strong correlation with nDIC and SST. From this calculation we obtained an empirical regression equation for Tahiti (R^2^=0.55, n=1423), Hawaii (R^2^=0.68, n=2253), and Marquesas (R^2^=0.65, n=3704) using the below parameters. Root mean squares of *ε* are 5.5, 5.4 and 6.4 μmol/Kg, respectively.

| Parameter | Tahiti | Hawaii | Marquesas |
| --- | --- | --- | --- |
| C_0_ | 1936.6 | 1956.0 | 1974.0 |
| C_1_ | 0.73 | 0.87 | 0.58 |
| C_2_ | -6.78 | -4.16 | -14.6 |
| C_3_ | 0.40 | 0.26 | -3.0 |
| C_4_ | -0.38 | -0.064 | 0.37 |
| C_5_ | -10.7 | -12.4 | -36.3 |
| T_ave_ (˚C) | 27.4 | 25.7 | 28.2 |

An example of multiple regression analysis for Tahiti is shown in Fig. S1. Seasonality in pH and ocean acidification from AD 1975 to 2000 is evident. Rates of ocean acidification are consistent with previous studies and consistent with a primary anthropogenic CO_2_ influence^71^.

We further assessed the validity of this estimation to compare the regression results for the Hawaii area with the HOT dataset^62,69^ (Fig. S2). The rate of pCO_2_ increase due to ocean acidification agrees well with that of the atmosphere except for the most recent interval. This may be due to biases derived from multiple regression analysis because SOCAT fCO_2_ data are heavily concentrated in the mid 1990s. pH and pCO_2_ estimation generally yield a slightly lower seasonality than that measured at the fixed station. However, the estimation reflects *in situ* observations well, considering the temporally and spatially wide distribution of shipboard measurements of the SOCAT datasets, various errors derived from SSS datasets and TA estimation, as well as DIC measurement precision.

We also calculated differences in pH and pCO_2_ between Tahiti and Marquesas using the same methodology (Fig. S3). As Marquesas is located closer to the equatorial upwelling zone than Tahiti, pH (pCO_2_) is lower (higher) by 0.04 (43.9 μatm). All calculations considered this offset. After detrending, seasonality of pH (pCO_2_) around Tahiti and Marquesas is estimated to be 0.018 (11.7 μatm) and 0.011 (13.0 μatm), respectively. δ^11^B data are unaffected by seasonality due to an average sample resolution of > 1 year^11,24,48^.

Multiple regression is useful to quantify ocean acidification, but results cannot be extrapolated beyond the instrumental record. If we apply equation (S2) for Tahiti to the preindustrial period (AD 1700s), assuming unchanged SST and SSS, pH of seawater is calculated to be ~8.45, which is inconsistent with previous estimations, e.g., “ca. 0.1 higher pH (thus ca. 8.2)” before the Industrial Revolution^20,21,72–76^. Therefore we estimated annually averaged pH variations after the Industrial Revolution using the method modified from Tans^76^, which uses an empirical pH estimation equation based on atmospheric pCO_2_, taking into account the reaction of borate with anthropogenic CO_2_;

$$pH=pH_{0}-0.85*log\frac{X}{280}$$

(S3)

Where X is atmospheric CO_2_ concentration obtained from *in situ* pCO_2_ observations at Mauna Loa^22^ and the Law Dome ice core^23^. pH_0_ is the late Holocene pH based on preindustrial atmospheric pCO_2_ (280 μatm) and is calculated based on annual average pH and atmospheric pCO_2_ at AD 1991 (8.111 and 355.6 μatm, respectively^22^ (Fig. S4). This yields a value of 8.203, which is slightly higher than the preindustrial pH calculated from GLODAP DIC and TA^21,72,73^, which are 8.188 and 8.169 when anthropogenic DIC (DIC_ant_) incorporation are 50 and 36.2 μmol/Kg, respectively (Fig. S4). Gridded DIC_ant_ by GLODAP at 17.5˚S, 149.5˚W is reported as 36.2 μmol/Kg (ref. 77).

However, it has been suggested that this value is lower than expected due to thermodynamic considerations^72^. Thus we adopted an average value for subtropical Pacific of 50 μmol/Kg and further modified equation (S3) in order to reconcile the discrepancy. The final pH value obtained for Tahiti was obtained with the following equation:

$$pH=8.184-0.70*log\frac{X}{280}$$

(S4)

Preindustrial pH at Tahiti is calculated to be 8.184. This agrees well with the pH estimation from multiple regression analysis for 1979 - 1998 (Fig. S4b), and the trend for the recent two decades (0.0014 yr^-1^) is also consistent with repeatedly measured pH along with the WOCE P06 line at 32˚S in subtropical South Pacific (0.0016 yr^-1^ for 1994 - 2008)^78^.

**SST effects on pH and pCO_2_ variability.**

We calculated pH using the modern annual mean SST after evaluating the effects of potential temperature change. Determination of past pH and pCO_2_ requires knowledge of paleo-SST because these parameters, as well as the dissociation constant of boric acid (pK_B_), are strongly temperature dependent^14,15,50,66,68^ such that a 10ºC decrease in temperature corresponds to an approximate 0.1 unit increase in pH. Temperature reconstructions^79,80^ from the tropical and subtropical Pacific indicate relatively little change from modern values within the temporal range of our data. A compilation^80^ of SST records obtained from marine sediments indicates an overall increase from LGM to present, without major reversals during the YD or HS1, and with a total change of 2˚C from 15 ka.

However, coral SST reconstructions indicate lower temperatures (2 - 4 ºC) during the last deglaciation and early Holocene at Tahiti^25,81,82^, Therefore, we recalculated pCO_2_ considering the coldest reported SST to estimate the maximum range of pCO_2_ change and evaluate the effect on our conclusions (Fig. S6). For this, we used SST results from IODP Exp 310 corals that indicate SST was cooler by 3.5˚C at 15.0 ka BP (HS1)^25^, 2.1˚C at 14.2 ka BP Bølling/Allerød (B/A)^82^, 3.4˚C at 12.4 ka BP (YD)^82^, 3.2˚C at 9.5 ka BP during the Holocene^81^. Because there are no LGM SST data from this region, we employed a 5˚C LGM change^83,84^. (Note that this does not consider the possibility of changes in either seawater Sr/Ca through time or Sr/Ca-SST sensitivity as discussed in previous studies^25,81,82,85^ and therefore represents maximum potential SST change.)

Deglacial air-sea disequilibrium in the coral ΔpCO_2_ (Fig. S6b) is clearly insensitive to a large potential SST decrease. Carbon dioxide emission from the equatorial Pacific, as well as anomalously higher pCO_2_ at ends of HS1 and the YD, persist (Fig. S6b,c). We also note that only a slight lowering of LGM SST (1 – 3˚C),　the accepted equatorial Pacific range^79,80^, results in an estimated LGM ΔpCO_2_ that is nearly identical to that of the Holocene (under modern SST conditions)^80^ (Fig. S6b). This implies CO_2_ equilibration persisted during these periods.

**Effect of Number of Samples**

We analyzed all available high-quality, pristine *Porites* fossil coral recovered during IODP Exp. 310. After removing the three most prominent low-pH events, the least extreme of which is 8.09, the mean baseline pH is ~8.18 (n=24; 1σ=0.024; Fig. S7). Given this variance, we performed a Monte Carlo simulation to explore whether our number of samples (27) is sufficient to resolve the expected millennial-scale pH variations. We first created a theoretical, annual pH series that covers a slightly longer interval of time (6,500) as our postglacial coral data (6,470 years) and contains two equal amplitude and duration low-pH events (Fig S8a). The baseline of the theoretical series is ~8.18, and the amplitude of each event is 8.09. Total event duration is 1,000 years, corresponding to the characteristic timescale of the overturning circulation. Within each event, peak values persist for only 600 years, approximately half the duration of the shortest of the two low-pH events expressed in the foraminifer data of Palmer and Pearson (ref. 10; Fig., 3a in the main text).

We performed 100 separate simulations to consider datasets of varying number of samples (n) from 1 to 100. For each value of n, the theoretical pH series was resampled 100,000 times, and the resulting series were analyzed to determine if both low-pH events were resolved. A series was accepted only when two conditions were met (Fig S8b): 1) Both events were sampled and distinguished by an intervening baseline value; and 2) the amplitude of both resampled low-pH events exceeded the baseline (~8.18) by 2 standard deviations (~8.13), accounting for the background variance in the coral pH data (1σ=0.024). The two low-pH events in the theoretical series are resolved in 94% of the resampled series when n is 27 (Fig S9).


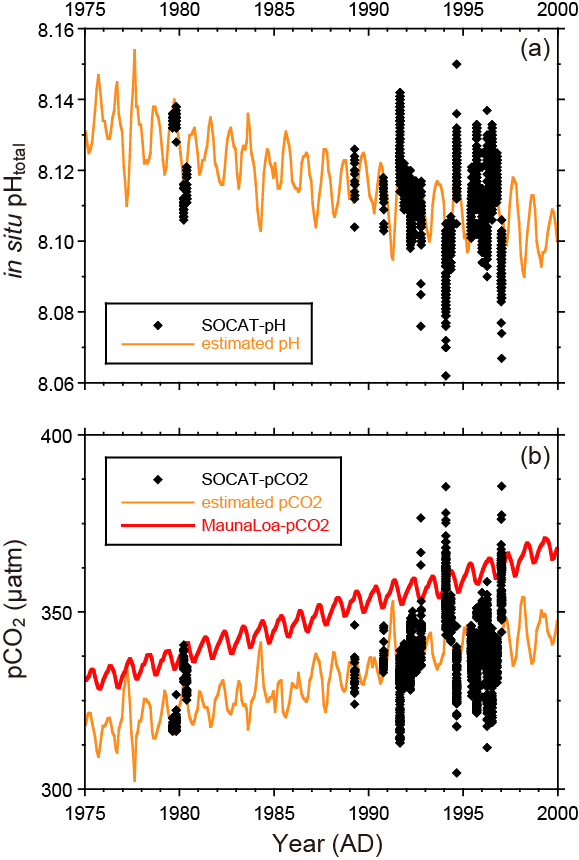


**Figure S1.** (**a**) *In situ* pH and (**b**) pCO_2_ calculated from SOCAT fCO_2_ (black diamonds) and estimated seasonal variations (yellow lines) using SODA SST and SSS for the years 1975 - 2000 around Tahiti. Atmospheric pCO_2_ continuously measured at Mauna Loa in Hawaii is also plotted in **b** (red line) (ref. 22).

**
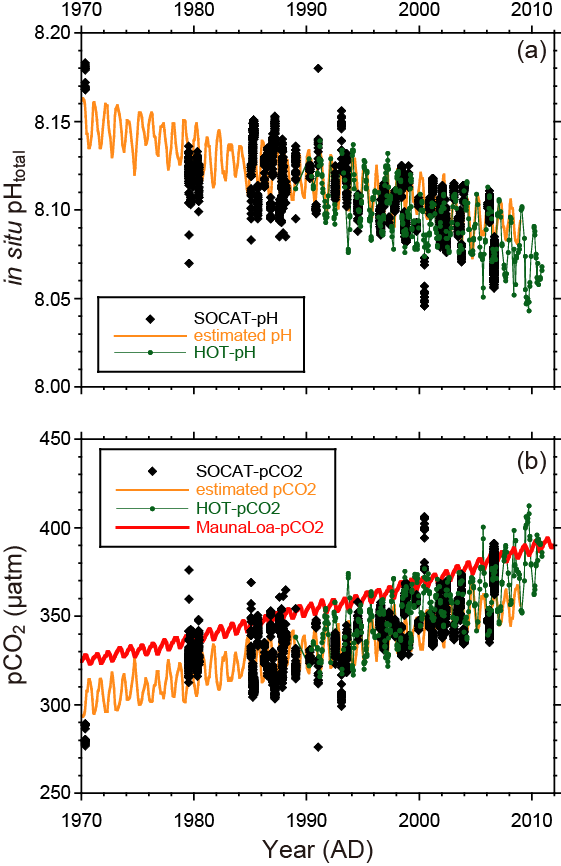
**

**Figure S2.** As in Fig. S1, but for Hawaii during 1970 - 2008**.** (**a**) *In situ* pH and (**b**) pCO_2_ variability and its comparison to HOT continuous measurements at Station ALOHA (22˚ 45'N, 158˚ 00'W; green dots with line) (ref. 62,69).

**
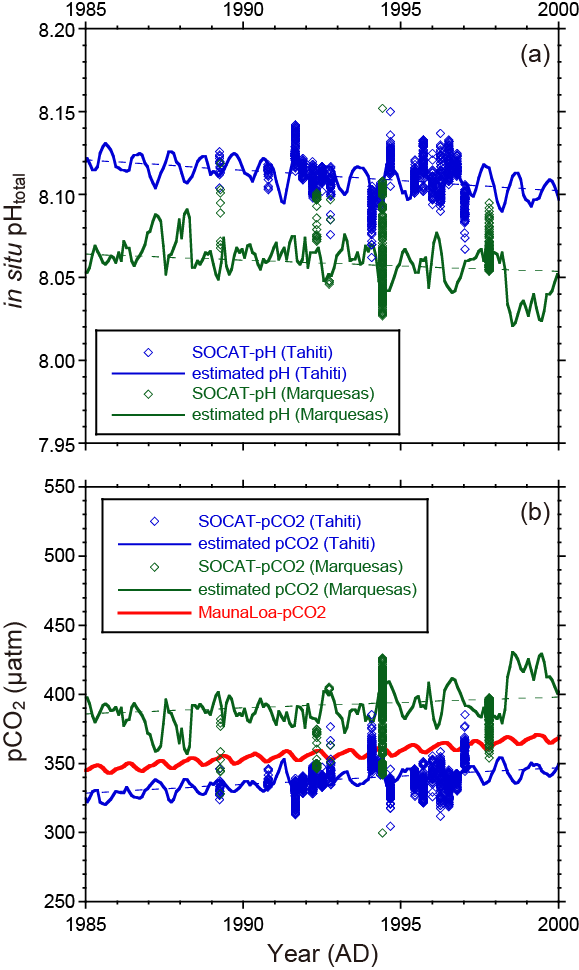
**

**Figure S3.** Comparison of *in situ* pH and pCO_2_ between Tahiti (blue) and Marquesas (green). (**a**) *In situ* pH and (**b**) pCO_2_ calculated from SOCAT fCO_2_ (open diamonds) and estimated seasonal variations (solid lines) using SODA SST and SSS from 1985 to 2000. Atmospheric pCO_2_ continuously measured at Mauna Loa in Hawaii is also plotted in **b** (red line) (ref. 22).

.

**
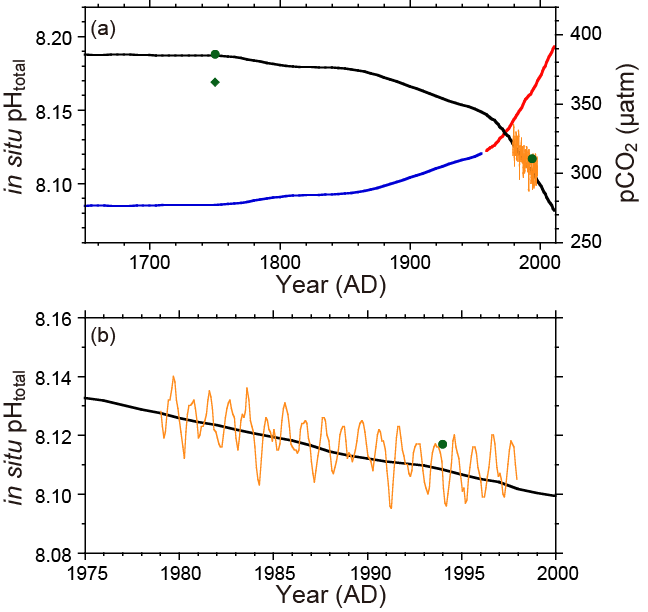
**

**Figure S4.** (**a**) pH around Tahiti and atmospheric pCO_2_ during 1650 - 2011. Seasonal (yellow) and annual (black) pH variations are estimated according to the methodology described in the Supplementary Methods. Green symbols are estimated pH at 1994 and preindustrial era from GLODAP data compilation (circle and diamond are calculated when anthropogenic DIC incorporation are 50 and 36.2 μmol/kg, respectively)^72,77^. Red and blue lines represent atmospheric pCO_2_ measured at Mauna Loa in Hawaii (annually averaged) (ref. 22) and that recovered from Law Dome ice core in Antarctica (5 years averaged) (ref. 23), respectively. (**b**) Enlarged view of pH during 1975 - 2000.

**
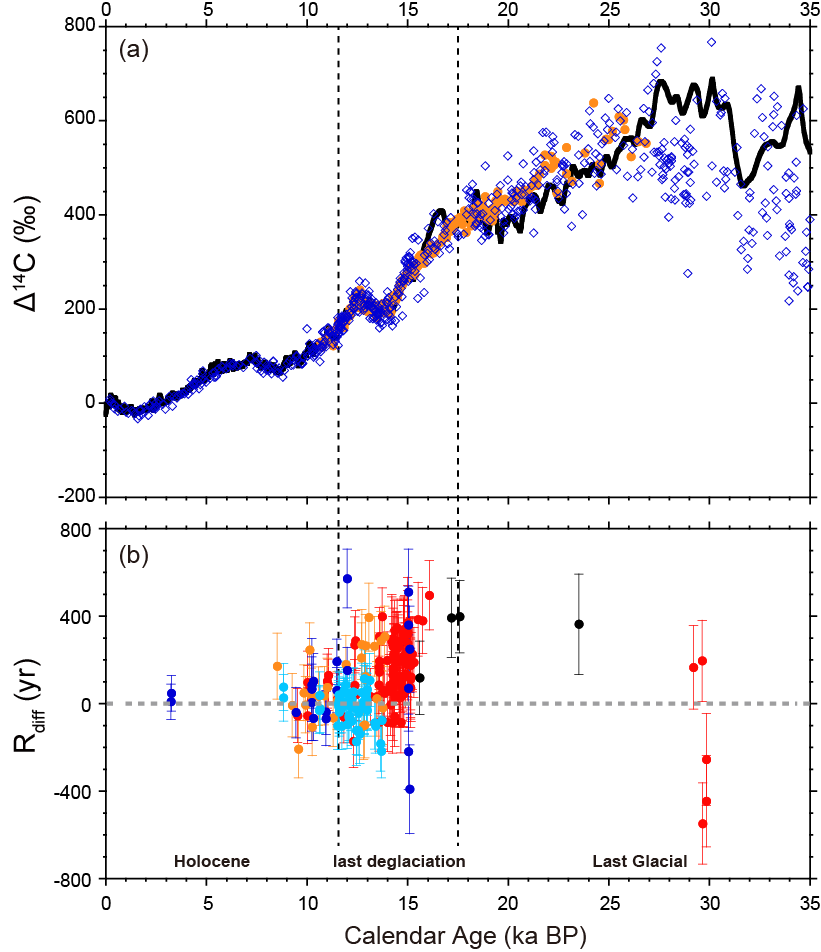
**

**Figure S5.** (**a**) Time series of atmoshperic Δ^14^C (black line: Intcal09 (ref. 56); blue diamonds: Lake Suigetsu^28^), DCF corrected Hulu cave speleothem^57^ (orange circles). (**b**) Differences between modern and past R around the equatorial Pacific Ocean. All data are from fossil corals (red: offshore Tahiti (this study); orange: reef crest of Tahiti barrier reef^58^, blue: Marquesas^26^; light blue: Kiritimati^59^; black: Mururoa^58^). Horizontal gray dashed line represents ‘R_diff_ = 0’. All R_diff_ data except for IODP Exp. 310 data that spans 29 - 30 ka were calculated using atmospheric Δ^14^C of INTCAL09 (for 29 - 30 ka, Lake Suigetsu data were used).

**
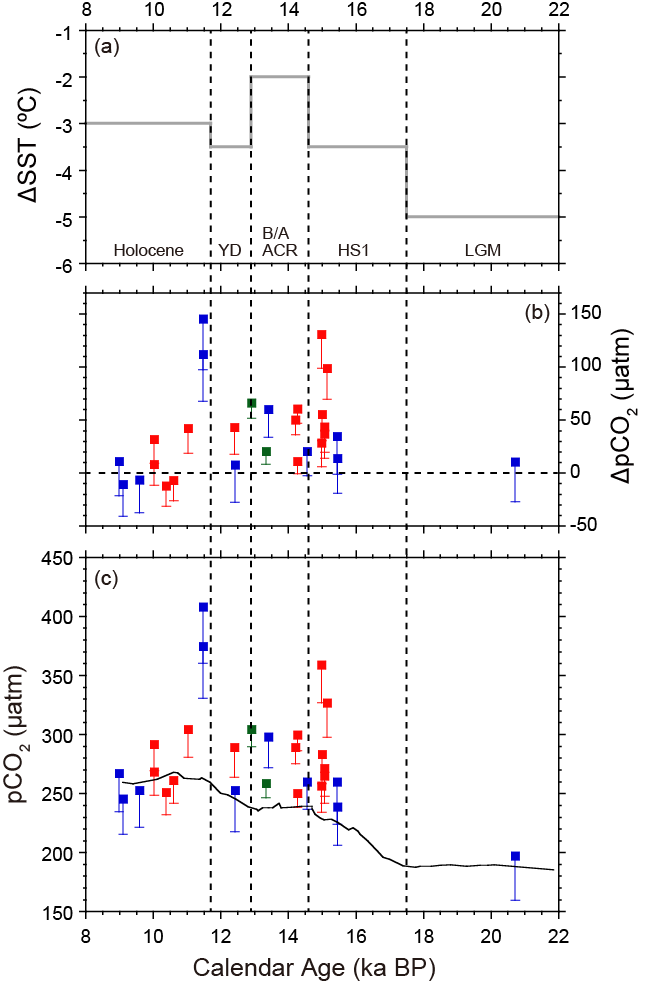
**

**Figure S6.** Evaluation of influence of SST change on pCO_2_ estimation. (**a**) Estimated SST differences compared to the preindustrial era using the most extreme potential decrease. The absolute minimum reported SST values from the equatorial Pacific were used for calculation of lower limits of uncertainty in **b** and **c**. (**b**) pCO_2_ difference between surface water at Tahiti and Marquesas and atmosphere. Horizontal dashed line represents ‘ΔpCO_2_ = 0’. (**c**) Calculated pCO_2_ of surface water around the equatorial South Pacific Ocean assuming the same SST to the present (legends is same as Figs. 3 and 4) and atmospheric pCO_2_ on the GICC05 timescale^1^ (black line).

**
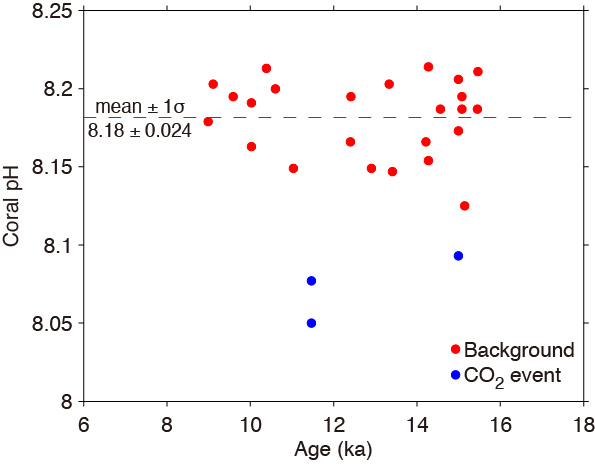
**

**Figure S7.** Coral pH data. Red indicates “baseline” values from which mean and standard deviation were calculated for use in the simulation. Blue values are low-pH events.


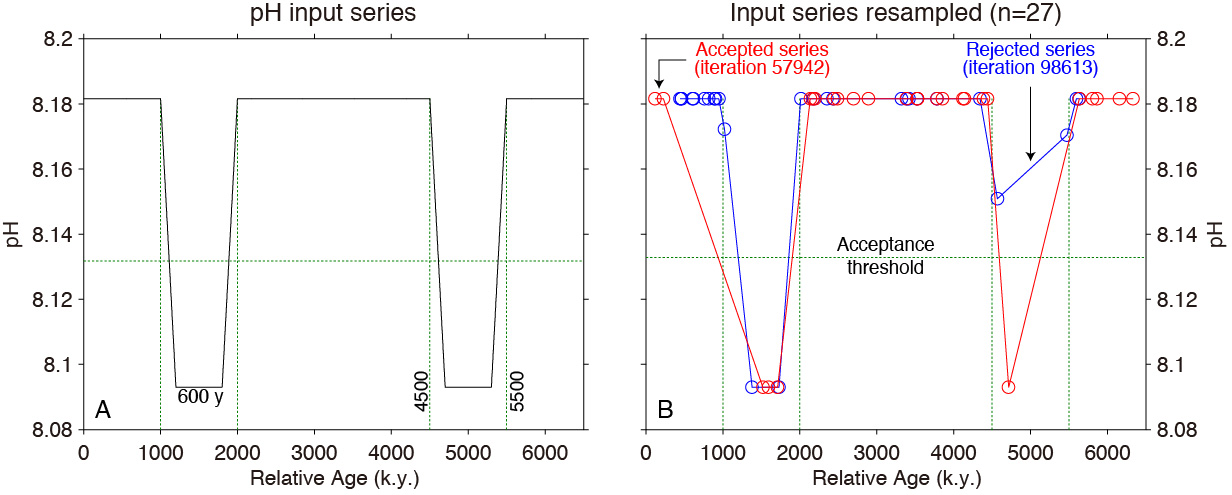


**Figure S8.** A) 6,500-year theoretical, annual pH input series with two millennial scale low-pH events. Horizontal green line indicates the 2σ threshold based on the variance in our coral pH data. B) An example of two (out of a total of 100,000) series of 27 samples. The accepted example (red, produced during iteration number 57,942) captures both low-pH events. While the rejected series (blue) also captures both events, the amplitude of the second event is indistinguishable from the background variance in the coral pH data at the 2σ level.


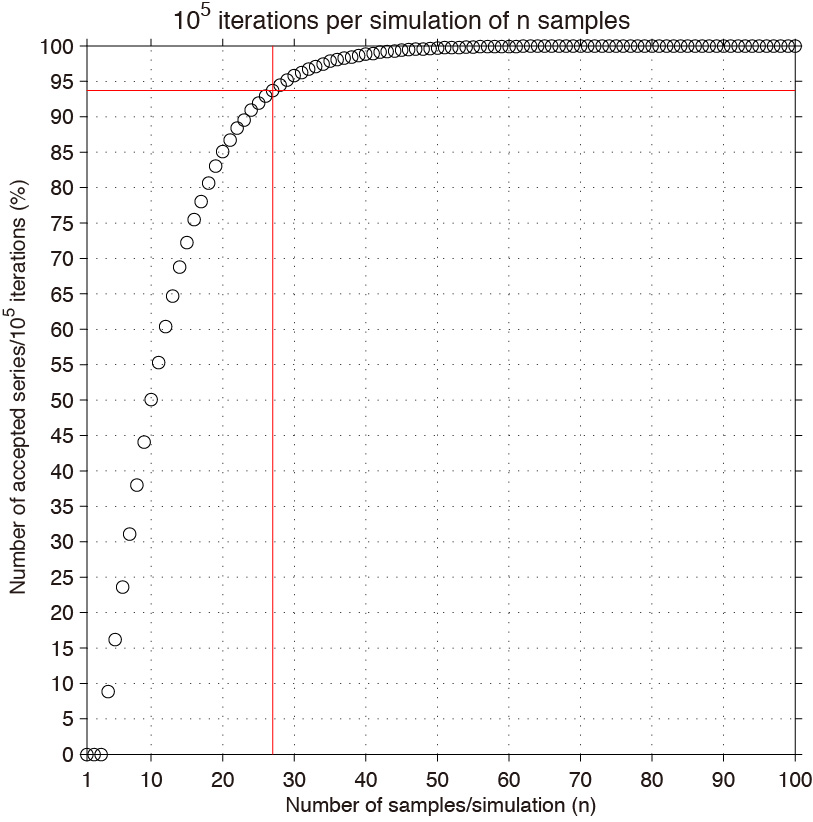


**Figure S9.** Results of simulating the minimum number of samples needed to reproduce the theoretical pH series. Red lines indicate n=27 (94%).

**Table S1. The δ^11^B values of *Porites* corals and calculated pH and pCO_2_**.

| **Location** | **^1^Sample ID 1** | **^2^Cal. age**  **[Years BP]** | **±1σ** | **Reference** | **δ^11^B**  **[‰]** | **^3^δ^11^B ave.**  **[‰]** | **±2σ** | **Reference** | **^4^pH** | **±2σ** | **pCO_2_**  **[μatm]** | **±2σ** | **ΔpCO_2_**  **[μatm]** |
| --- | --- | --- | --- | --- | --- | --- | --- | --- | --- | --- | --- | --- | --- |
| ***Fossil*** |  |  |  |  |  |  |  |  |  |  |  |  |  |
| Tahiti | 310-M0005B-3R-1W_58-67  (3116500) | 10387 | 74 | 48 | 26.29  26.32 | 26.31 | 0.18 | This study | 8.213 | 0.013 | 251 | 11 | -12 |
| Tahiti | 310-M0005C-8R-2W_0-5  (3116758) | 10608 | 52 | 48 | 26.13  26.12 | 26.12 | 0.18 | This study | 8.200 | 0.014 | 261 | 10 | -7 |
| Tahiti | 310-M0007A-18R-1 W _76-90  (3113922) | 10030 | 10 | 17,86 | 26.04  25.98 | 26.01 | 0.18 | This study | 8.191 | 0.014 | 268 | 11 | 8 |
| Tahiti | 310-M0007A-18R-1W_28-58  (3113876) | 10030 | 10 | 17,86 | 25.64  25.68 | 25.66 | 0.18 | This study | 8.163 | 0.014 | 292 | 13 | 32 |
| Tahiti | 310-M0007B-21R-1W_0-20  (3114984) | 11035 | 28 | 17,87 | 25.49  25.47 | 25.48 | 0.18 | This study | 8.149 | 0.014 | 304 | 13 | 42 |
| Tahiti | 310-M0018A-18R-1W_40-50  (3125580) | 14273 | 31.5 | 86 | 26.39  26.24 | 26.31 | 0.18 | This study | 8.214 | 0.013 | 250 | 10 | 11 |
| Tahiti | 310-M0018A-18R-1W_50-63  (3125582) | 14273 | 31.5 | 86 | 25.51  25.56 | 25.54 | 0.18 | This study | 8.154 | 0.014 | 300 | 12 | 61 |
| Tahiti | 310-M0009D-7R-1W_11-28  (3105506) | 14217 | 36 | 17,82,86 | 25.73  25.65 | 25.69 | 0.18 | This study | 8.166 | 0.014 | 289 | 12 | 50 |
| Tahiti | 310-M0023A-6R-1W_48-62  (3101822) | 12404 | 49 | 82 | 25.58  25.80 | 25.69 | 0.18 | This study | 8.166 | 0.014 | 289 | 12 | 43 |
| Tahiti | 310-M0024A-11R-1W_77-90  (3111904) | 14994 | 12.5 | 17,86 | 26.20  26.23 | 26.21 | 0.18 | This study | 8.206 | 0.014 | 256 | 11 | 28 |
| Tahiti | 310-M0024A-11R-1W_60-75  (3111884) | 14994 | 12.5 | 17,86 | 24.82  24.77 | 24.79 | 0.18 | This study | 8.093 | 0.015 | 359 | 15 | 131 |
| Tahiti | 310-M0024A-11R-2W_25-61  (3111958) | 14997 | 25 | 17,25,86 | 25.82  25.73 | 25.78 | 0.18 | This study | 8.173 | 0.014 | 283 | 12 | 55 |
| Tahiti | 310-M0024A-12R-2W_140-150  (3112030) | 15080 | 29 | 17 | 26.10  25.82 | 25.96 | 0.18 | This study | 8.187 | 0.014 | 271 | 11 | 43 |
| Tahiti | 310-M0024A-12R-2W_62-80  (3112024) | 15075 | 29 | 17 | 26.04  26.09 | 26.07 | 0.18 | This study | 8.195 | 0.014 | 265 | 11 | 37 |
| Tahiti | 310-M0024A-13R-1W_32-41  (3112052) | 15149 | 15.5 | 17,86 | 25.14  25.21 | 25.18 | 0.18 | This study | 8.125 | 0.015 | 327 | 13 | 99 |
| Tahiti | Ta P8-348 | 12910 | 30 | 88 |  | 25.9 | 0.25 | 11 | 8.149 | 0.020 | 304 | 18 | 66 |
| Tahiti | Ta P8-353 | 13335 | 30 | 88 |  | 26.6 | 0.25 | 11 | 8.203 | 0.019 | 259 | 15 | 21 |
| Marquesas | Eiao DR16(3) | 8990 | 130 | 89 |  | 26.0 | 0.25 | 11 | 8.179 | 0.020 | 267 | 18 | 11 |
| Marquesas | Eiao DR16(5) | 9110 | 130 | 89 |  | 26.3 | 0.25 | 11 | 8.203 | 0.019 | 245 | 16 | -11 |
| Marquesas | Eiao DR12(1) | 9590 | 180 | 89 |  | 26.2 | 0.25 | 11 | 8.195 | 0.019 | 253 | 16 | -6 |
| Marquesas | DW1281 75a2 | 11470 | 90 | 26 |  | 24.8 | 0.25 | 11 | 8.077 | 0.022 | 375 | 27 | 112 |
| Marquesas | DW1281 75a2 | 11470 | 90 | 26 |  | 24.5 | 0.25 | 11 | 8.050 | 0.023 | 408 | 29 | 145 |
| Marquesas | Hiva Oa DR10(2) | 12420 | 100 | 89 |  | 26.2 | 0.25 | 11 | 8.195 | 0.019 | 253 | 16 | 8 |
| Marquesas | Eiao DR11bis(4) | 13410 | 190 | 89 |  | 25.6 | 0.25 | 11 | 8.147 | 0.021 | 298 | 20 | 60 |
| Marquesas | Eiao DR8(1) | 14560 | 180 | 89 |  | 26.1 | 0.25 | 11 | 8.187 | 0.020 | 260 | 18 | 21 |
| Marquesas | Hiva Oa DR14bis(1) | 15450 | 150 | 89 |  | 26.1 | 0.25 | 11 | 8.187 | 0.020 | 260 | 18 | 35 |
| Marquesas | Hiva Oa DR8bis(1) | 15460 | 110 | 89 |  | 26.4 | 0.25 | 11 | 8.211 | 0.019 | 239 | 16 | 14 |
| Marquesas | Hiva OaDR5 | 20720 | 200 | 89 |  | 27.1 | 0.25 | 11 | 8.263 | 0.018 | 197 | 14 | 10 |
| ***Modern*** |  |  |  |  |  |  |  |  |  |  |  |  |  |
| Moorea | COM2 | - (AD1991) |  | 11 |  | 25.3 | 0.30 | 24 | 8.096 | 0.025 | 356 | 25 | -15 |
| Moorea | MOO 3A-1-02 | - (AD1950) |  | 11 |  | 25.8 | 0.25 | 11 | 8.145 | 0.023 | 308 | 20 | -10 |
| Marquesas | Nuku Hiva DR6(1) | 250 (AD1700) | 30 | 89 |  | 26.2 | 0.25 | 11 | 8.205 | 0.022 | 244 | 18 | -17 |

(1) Original sample code of IODP Exp. 310 and sample code in individual laboratories.

(2) Calendar age of fossil corals. For 310-M0005B-3R-1W_58-67 and 310-M0005C-8R-2W_0-5, dating was conducted by ^14^C dating method^48^.

(3) Boron isotope values for this study are average values of duplicate analysis. Those for Douville *et al*.^11^ are mainly of replicate analysis. See Douville *et al*.^11^ for details.

(4) pH for Marquesas were added by 0.04 after calculation for comparison. pK_B_ for Tahiti-Moorea and Marquesas are 8.57 (SST = 27.4 ºC; SSS = 35.9) and 8.56 (SST = 27.9 ºC; SSS = 35.6), respectively.

Table S2. Compiled radiocarbon and U/Th ages and calculated marine reservoir ages.

| **Core ID** | **Core depth** | **^1^Conv. ^14^C age**  **[Years]** | **±1σ** | **Reference** | **U/Th (Cal. age)**  **[Years BP]** | **±2σ** | **Reference** | **R**  **[Years]** | **^2^R_diff_**  **[Years]** | **±1σ** |
| --- | --- | --- | --- | --- | --- | --- | --- | --- | --- | --- |
| 310-M0005A-12R-1W | 51-54 | 9885 | 35 | 86 | 11032 | 20.0 | 17,86 | 322 | 87 | 117 |
| 310-M0005C-11R-1W | 46-59 | 10370 | 35 | 86 | 11837 | 25.0 | 17,86 | 175 | -60 | 118 |
| 310-M0005D-2R-1W | 107-115 | 10780 | 35 | 86 | 12430 | 30.0 | 17,86 | 278 | 43 | 118 |
| 310-M0005D-5R-2W | 0-5 | 11545 | 35 | 86 | 13162 | 40.0 | 17,86 | 267 | 32 | 129 |
| 310-M0005D-6R-2W | 0-5 | 12230 | 40 | 86 | 13795 | 31.0 | 17,86 | 296 | 61 | 135 |
| 310-M0007A-18R-1W | 28-58 | 9214 | 89 | 48 | 10030 | 20.0 | 17,86 | 333 | 98 | 143 |
| 310-M0007A-18R-1W | 76-90 | 9175 | 30 | 86 | 10030 | 18.0 | 17,86 | 294 | 59 | 115 |
| 310-M0007A-18R-1W | 76-90 | 9062 | 65 | 48 | 10030 | 20.0 | 17,86 | 181 | -54 | 129 |
| 310-M0007B-11R-2W | 0-14 | 8690 | 50 | 90 | 9523 | 33.0 | 81 | 181 | -54 | 123 |
| 310-M0007B-21R-1W | 0-20 | 9917 | 48 | 48 | 11010 | 40.0 | 87 | 338 | 103 | 123 |
| 310-M0007B-21R-1W | 0-20 | 9917 | 48 | 48 | 11060 | 40.0 | 17 | 365 | 130 | 123 |
| 310-M0009A-6R-1W | 38-48 | 12550 | 60 | 86 | 14240 | 30.0 | 17,86 | 147 | -88 | 139 |
| 310-M0009B-13R-1W | 11-18 | 12930 | 50 | 86 | 14520 | 20.0 | 17,86 | 505 | 270 | 136 |
| 310-M0009B-14R-1W | 22-25 | 13160 | 50 | 86 | 15148 | 20.0 | 17,86 | 355 | 120 | 182 |
| 310-M0009B-15R-1W | 13-20 | 13880 | 50 | 86 | 16081 | 60.0 | 17,86 | 731 | 496 | 158 |
| 310-M0009B-9R-2W | 0-5 | 12580 | 50 | 86 | 14349 | 22.0 | 17,86 | 176 | -59 | 136 |
| 310-M0009B-9R-2W | 0-5 | 12665 | 40 | 86 | 14349 | 22.0 | 17,86 | 261 | 26 | 133 |
| 310-M0009C-17R-2W | 0-10 | 13610 | 50 | 86 | 15511 | 30.0 | 17,86 | 621 | 386 | 168 |
| 310-M0009C-6R-1W | 38-43 | 12300 | 50 | 86 | 13849 | 29.0 | 17,86 | 283 | 48 | 136 |
| 310-M0009D-10R-2W | 74-78 | 13050 | 50 | 86 | 14790 | 30.0 | 17 | 551 | 316 | 154 |
| 310-M0009D-10R-2W | 96-107 | 13030 | 50 | 86 | 14789 | 26.0 | 17,86 | 532 | 297 | 154 |
| 310-M0009D-10R-2W | 96-107 | 13050 | 50 | 86 | 14789 | 26.0 | 17,86 | 552 | 317 | 154 |
| 310-M0009D-11R-1W | 13-26 | 12985 | 40 | 86 | 14916 | 35.0 | 17,86 | 435 | 200 | 153 |
| 310-M0009D-7R-1W | 11-28 | 12904 | 156 | 48 | 14211 | 39.0 | 82 | 531 | 296 | 203 |
| 310-M0009D-7R-1W | 11-28 | 12680 | 40 | 86 | 14211 | 39.0 | 82 | 307 | 72 | 134 |
| 310-M0009D-7R-1W | 11-28 | 12904 | 156 | 48 | 14223 | 60.0 | 17,86 | 519 | 284 | 203 |
| 310-M0009D-7R-1W | 11-28 | 12680 | 40 | 86 | 14223 | 60.0 | 17,86 | 294 | 59 | 135 |
| 310-M0009D-9R-1W | 66-77 | 12840 | 50 | 86 | 14490 | 33.0 | 17,86 | 427 | 192 | 137 |
| 310-M0009D-9R-1W | 99-103 | 12950 | 45 | 86 | 14530 | 50.0 | 17,86 | 520 | 285 | 135 |
| 310-M0009E-7R-1W | 5-13 | 12585 | 50 | 86 | 14116 | 43.0 | 86 | 319 | 84 | 141 |
| 310-M0009E-9R-1W | 32-36 | 12845 | 40 | 86 | 14360 | 40.0 | 17,86 | 441 | 206 | 133 |
| 310-M0009E-9R-1W | 69-73 | 12775 | 40 | 86 | 14770 | 40.0 | 17,86 | 278 | 43 | 151 |
| 310-M0015A-33R-1W | 29-40 | 12120 | 35 | 86 | 13577 | 23.0 | 17,86 | 403 | 168 | 128 |
| 310-M0015A-33R-1W | 29-40 | 12270 | 50 | 86 | 13590 | 35.0 | 17,86 | 542 | 307 | 133 |
| 310-M0015A-36R-1W | 51-52 | 12925 | 45 | 86 | 14419 | 30.0 | 17,86 | 521 | 286 | 132 |
| 310-M0015A-36R-2W | 0-6 | 12915 | 50 | 86 | 14518 | 20.0 | 17,86 | 492 | 257 | 136 |
| 310-M0015A-37R-1W | 19-28 | 12765 | 40 | 86 | 14650 | 20.0 | 17,86 | 266 | 31 | 135 |
| 310-M0015A-37R-1W | 19-28 | 12830 | 40 | 86 | 14650 | 20.0 | 17,86 | 331 | 96 | 135 |
| 310-M0016A-36R-2W | 5-10 | 12810 | 40 | 86 | 14558 | 24.0 | 17,86 | 361 | 126 | 130 |
| 310-M0016A-36R-2W | 5-10 | 12790 | 45 | 86 | 14558 | 24.0 | 17,86 | 341 | 106 | 132 |
| 310-M0018A-18R-1W | 40-50 | 12759 | 58 | 48 | 14273 | 63.0 | 86 | 332 | 97 | 141 |
| 310-M0018A-18R-1W | 40-50 | 12825 | 45 | 86 | 14273 | 63.0 | 86 | 398 | 163 | 136 |
| 310-M0018A-18R-1W | 40-50 | 12740 | 45 | 86 | 14273 | 63.0 | 86 | 313 | 78 | 136 |
| 310-M0018A-18R-1W | 50-63 | 12712 | 175 | 48 | 14273 | 63.0 | 86 | 285 | 50 | 217 |
| 310-M0018A-19R-1W | 107-110 | 12845 | 40 | 86 | 14338 | 27.0 | 86 | 438 | 203 | 133 |
| 310-M0018A-7R-1W | 73-82 | 10280 | 35 | 86 | 11488 | 29.0 | 86 | 257 | 22 | 118 |
| 310-M0020A-16R-1W | 55-66 | 11995 | 40 | 86 | 13724 | 57.0 | 86 | 160 | -75 | 132 |
| 310-M0020A-21R-2W | 13-20 | 12530 | 40 | 86 | 14145 | 45.0 | 86 | 231 | -4 | 141 |
| 310-M0020A-23R-1W | 56-64 | 12925 | 40 | 86 | 14450 | 59.0 | 86 | 522 | 287 | 135 |
| 310-M0020A-23R-1W | 56-64 | 12840 | 50 | 86 | 14450 | 59.0 | 86 | 437 | 202 | 138 |
| 310-M0020A-23R-2W | 72-78 | 12815 | 50 | 86 | 14734 | 57.0 | 86 | 309 | 74 | 150 |
| 310-M0020A-24R-2W | 38-42 | 12655 | 40 | 86 | 14663 | 61.0 | 86 | 149 | -86 | 138 |
| 310-M0021A-13R-2W | 66-75 | 12320 | 50 | 86 | 14015 | 40.0 | 17,86 | 150 | -85 | 134 |
| 310-M0021B-16R-1W | 39-44 | 12975 | 50 | 86 | 14350 | 22.0 | 17,86 | 570 | 335 | 136 |
| 310-M0023A-11R-1W | 22-31 | 11930 | 40 | 53,86 | 13460 | 20.0 | 17,86 | 268 | 33 | 136 |
| 310-M0023A-11R-2W | 112-121 | 12035 | 50 | 53 | 13570 | 20.0 | 17 | 321 | 86 | 132 |
| 310-M0023A-12R-1W | 140-144 | 12490 | 40 | 86 | 13738 | 18.0 | 17,86 | 634 | 399 | 131 |
| 310-M0023A-12R-1W | 32-38 | 12100 | 40 | 86 | 13580 | 20.0 | 17,86 | 380 | 145 | 129 |
| 310-M0023A-12R-1W | 32-38 | 12150 | 40 | 53 | 13580 | 20.0 | 17,86 | 430 | 195 | 129 |
| 310-M0023A-13R-2W | 32-37 | 12885 | 50 | 53 | 14310 | 40.0 | 17 | 464 | 229 | 137 |
| 310-M0023A-13R-2W | 32-37 | 12885 | 50 | 86 | 14312 | 38.0 | 17,86 | 462 | 227 | 137 |
| 310-M0023A-14R-1W | 0-20 | 12750 | 40 | 86 | 14589 | 25.0 | 17,86 | 277 | 42 | 131 |
| 310-M0023A-5R-1W | 45-52 | 10695 | 35 | 86 | 12370 | 40.0 | 17,86 | 319 | 84 | 118 |
| 310-M0023A-5R-1W | 92-103 | 10880 | 60 | 53 | 12370 | 36.0 | 86 | 504 | 269 | 128 |
| 310-M0023A-6R-1W | 48-62 | 10968 | 74 | 48 | 12404 | 49.0 | 82 | 524 | 289 | 136 |
| 310-M0023B-12R-1W | 30-33 | 12575 | 35 | 86 | 13989 | 16.0 | 17,86 | 434 | 199 | 128 |
| 310-M0023B-12R-2W | 113-127 | 12790 | 50 | 53 | 14278 | 15.0 | 86 | 366 | 131 | 135 |
| 310-M0023B-12R-2W | 113-127 | 12810 | 50 | 86 | 14278 | 15.0 | 86 | 386 | 151 | 135 |
| 310-M0023B-12R-2W | 113-127 | 12790 | 50 | 86 | 14278 | 15.0 | 86 | 366 | 131 | 135 |
| 310-M0023B-12R-2W | 113-127 | 12790 | 50 | 86 | 14285 | 25.0 | 17 | 369 | 134 | 136 |
| 310-M0023B-12R-2W | 113-127 | 12810 | 50 | 86 | 14285 | 25.0 | 17 | 389 | 154 | 136 |
| 310-M0023B-12R-2W | 113-127 | 12790 | 50 | 53 | 14285 | 25.0 | 17 | 369 | 134 | 136 |
| 310-M0023B-15R-1W | 0-5 | 12925 | 50 | 86 | 14282 | 30.0 | 17,86 | 497 | 262 | 136 |
| 310-M0023B-15R-1W | 0-5 | 12960 | 60 | 86 | 14282 | 30.0 | 17,86 | 532 | 297 | 140 |
| 310-M0024A-10R-1W | 65-75 | 12730 | 50 | 86 | 14581 | 52.0 | 17,86 | 263 | 28 | 135 |
| 310-M0024A-10R-1W | 98-116 | 12920 | 70 | 86 | 14609 | 26.0 | 86 | 436 | 201 | 144 |
| 310-M0024A-10R-2W | 69-72 | 12935 | 40 | 86 | 14749 | 30.0 | 17,86 | 437 | 202 | 148 |
| 310-M0024A-10R-2W | 69-72 | 12850 | 50 | 53,86 | 14749 | 30.0 | 17,86 | 352 | 117 | 151 |
| 310-M0024A-11R-1W | 60-75 | 13082 | 90 | 48 | 14994 | 25.0 | 17,25,86 | 475 | 240 | 179 |
| 310-M0024A-11R-1W | 77-90 | 13160 | 161 | 48 | 14994 | 25.0 | 17,25,86 | 553 | 318 | 224 |
| 310-M0024A-11R-2W | 1-62 | 13025 | 40 | 53,86 | 14997 | 50.0 | 17,25,86 | 415 | 180 | 162 |
| 310-M0024A-11R-2W | 1-62 | 13121 | 121 | 48 | 14997 | 50.0 | 17,25,86 | 511 | 276 | 198 |
| 310-M0024A-11R-2W | 73-89 | 13050 | 70 | 90 | 14997 | 50.0 | 17,25,86 | 440 | 205 | 172 |
| 310-M0024A-12R-2W | 140-150 | 13173 | 104 | 48 | 15000 | 50.0 | 17 | 563 | 328 | 189 |
| 310-M0024A-12R-2W | 140-150 | 13173 | 104 | 48 | 15159 | 30.0 | 17 | 357 | 122 | 204 |
| 310-M0024A-12R-2W | 62-80 | 13217 | 131 | 48 | 15000 | 50.0 | 17 | 607 | 372 | 205 |
| 310-M0024A-12R-2W | 62-80 | 13217 | 131 | 48 | 15149 | 30.0 | 17,86 | 411 | 176 | 219 |
| 310-M0024A-13R-1W | 32-41 | 13210 | 115 | 48 | 15149 | 31.0 | 17,86 | 418 | 183 | 211 |
| 310-M0024A-13R-1W | 32-41 | 13100 | 40 | 86 | 15149 | 31.0 | 17,86 | 308 | 73 | 181 |
| 310-M0024A-13R-1W | 32-41 | 13140 | 50 | 86 | 15149 | 31.0 | 17,86 | 348 | 113 | 183 |
| 310-M0024A-14R-1W | 24-28 | 13378 | 55 | 48 | 15223 | 40.0 | 17,86 | 461 | 226 | 172 |
| 310-M0024A-14R-1W | 24-28 | 13385 | 45 | 86 | 15223 | 40.0 | 17,86 | 462 | 227 | 162 |
| 310-M0024A-15R-1W | 16-20 | 13700 | 40 | 86 | 15742 | 30.0 | 17,86 | 615 | 380 | 152 |
| 310-M0024A-1R-1W | 36-41 | 10445 | 40 | 53 | 12310 | 30.0 | 17 | 64 | -171 | 120 |
| 310-M0024A-4R-1W | 137-141 | 11860 | 40 | 53 | 13560 | 40.0 | 17 | 149 | -86 | 130 |
| 310-M0025A-10R-1W | 40-46 | 12990 | 35 | 86 | 14478 | 24.0 | 86 | 582 | 347 | 132 |
| 310-M0025A-10R-1W | 40-46 | 12845 | 50 | 86 | 14478 | 24.0 | 86 | 437 | 202 | 136 |
| 310-M0025B-10R-1W | 0-5 | 12910 | 60 | 86 | 14901 | 22.0 | 17,86 | 372 | 137 | 155 |
| 310-M0025B-10R-1W | 14-22 | 12990 | 50 | 53,86 | 14900 | 20.0 | 17 | 453 | 218 | 151 |
| 310-M0025B-11R-1W | 70-74 | 13410 | 60 | 86 | 15310 | 23.0 | 17,86 | 424 | 189 | 160 |
| 310-M0025B-9R-2W | 60-70 | 13060 | 50 | 86 | 14801 | 32.0 | 17,86 | 559 | 324 | 153 |
| 310-M0025B-9R-2W | 60-70 | 12955 | 40 | 86 | 14801 | 32.0 | 17,86 | 454 | 219 | 150 |
| 310-M0025B-9R-2W | 60-70 | 13075 | 35 | 86 | 14801 | 32.0 | 17,86 | 574 | 339 | 149 |
| 310-M0025B-9R-2W | 60-70 | 12785 | 50 | 86 | 14801 | 32.0 | 17,86 | 284 | 49 | 153 |
| 310-M0025B-9R-2W | 60-70 | 13070 | 35 | 86 | 14801 | 32.0 | 17,86 | 569 | 334 | 149 |
| 310-M0026A-5R-1W | 4-18 | 12935 | 45 | 86 | 14720 | 25.0 | 17,86 | 431 | 196 | 144 |
| 310-M0026A-5R-1W | 117-127 | 13080 | 80 | 86 | 14852 | 30.0 | 17,86 | 570 | 335 | 161 |
| 310-M0009B-16R-2W | 13-17 | 25970 | 100 | 86 | 29631 | 62.0 | 86 | 431 | 196 | 186 |
| 310-M0009B-17R-1W | 5-10 | 25530 | 140 | 86 | 29838 | 53.0 | 86 | -210 | -445 | 209 |
| 310-M0009B-17R-1W | 5-10 | 25720 | 140 | 86 | 29838 | 53.0 | 86 | -20 | -255 | 209 |
| 310-M0009B-17R-1W | 70-80 | 25260 | 100 | 86 | 29666 | 58.0 | 86 | -313 | -548 | 185 |
| 310-M0009D-14R-2W | 81-90 | 25530 | 110 | 86 | 29209 | 51.0 | 86 | 401 | 166 | 190 |

(1) AMS ^14^C ages (radiocarbon years) are calculated using a half time of 5730-years without any marine carbon reservoir age correction.

(2) A difference of marine reservoir ages from modern one.

**Supplementary References**

1. Pfeil, B. Surface Ocean CO_2_ Atlas (SOCAT). <http://www.socat.info/access.html>, (2012) Date of access:11/12/2012.
2. Dore, J. E., Lukas, R., Sadler, D. W., Church, M. J. & Karl, D. M. Physical and biogeochemical modulation of ocean acidification in the central North Pacific. *Proc. Natl. Acad. Sci.* *USA* **106**, 12235–12240 (2009).
3. Bates, N. R. Interannual variability of the oceanic CO_2_ sink in the subtropical gyre of the North Atlantic Ocean over the last 2 decades. *J. Geophys. Res.* **112**, JC003759 (2007).
4. Lee, K. *et al*. Global relationships of total alkalinity with salinity and temperature in surface waters of the world’s oceans. *Geophys. Res. Lett.* **33**, GL027207 (2006).
5. Carton, J. A. & Giese, B. S., CARTON-GIESE SODA 2.1.6. <http://iridl.ldeo.columbia.edu/SOURCES/.CARTON-GIESE/.SODA/.v2p1p6/>, (2012) Date of access: 20/12/2012)
6. Robbins, L. L., Hansen, M. E., Kleypas, J. A. & Meylan, S. C. CO2calc—A user-friendly seawater carbon calculator for Windows, Max OS X, and iOS (iPhone). *U.S. Geological Survey Open-File Report* 2010–1280 (2010).
7. Lueker, T. J., Dickson, A. G. & Keeling, C. D. Ocean pCO_2_ calculated from dissolved inorganic carbon, alkalinity, and equations for K_1_ and K_2_: validation based on laboratory measurements of CO_2_ in gas and seawater at equilibrium. *Mar. Chem.* **70**, 105–119 (2000).
8. Dickson, A. G. Thermodynamics of the dissociation of boric acid in synthetic seawater from 273.15 to 318.15 K. *Deep-Sea Res.* **37**, 755–766 (1990).
9. Fujieki, L. A., HOT : the Hawaii Ocean Time–Series. <http://hahana.soest.hawaii.edu/hot/hot_jgofs.html>, (2012) Date of access: 25/12/2012
10. Ishii, M. *et al*. Ocean acidification off the south coast of Japan: A result from time series observations of CO_2_ parameters from 1994 to 2008. *J. Geophys. Res.* **116**, JC006831 (2011).
11. Lenton, A. *et al*. The observed evolution of oceanic pCO_2_ and its drivers over the last two decades. *Glob. Biogeochem. Cycles* **26**, GB004095 (2012).
12. Sabine, C. L. *et al*. Distribution of anthropogenic CO_2_ in the Pacific Ocean. *Glob. Biogeochem. Cycles* **16**, GB001639 (2002).
13. Sabine, C. L. *et al*. The Oceanic Sink for Anthropogenic CO_2_. *Science* **305**, 367–371 (2004).
14. Orr, J. C. *et al*. Anthropogenic ocean acidification over the twenty-first century and its impact on calcifying organisms. *Nature* **437**, 681–686 (2005).
15. Hönisch, B. & Hemming, N. G. Surface ocean pH response to variations in pCO_2_ through two full glacial cycles. *Earth Planet. Sci. Lett.* **236**, 305–314 (2005).
16. Tans, P. An Accounting of the Observed Increase in Oceanic and Atmospheric CO_2_ and an Outlook for the Future. *Oceanography* **22**, 26–35 (2009).
17. Kozyr, A., GLODAP Carbon Synthesis Project. <http://cdiac.ornl.gov/oceans/glodap/>, (2010) Date of access:14/01/2013
18. Waters, J. F., Millero, F. J. & Sabine, C. L. Changes in South Pacific anthropogenic carbon. *Glob. Biogeochem. Cycles* **25**, GB003988 (2011).
19. MARGO Project Members Constraints on the magnitude and patterns of ocean cooling at the Last Glacial Maximum. *Nature Geosci.* **2**, 127–132 (2009).
20. Kiefer, T. & Kienast, M. Patterns of deglacial warming in the Pacific Ocean: a review with emphasis on the time interval of Heinrich event 1. *Quat. Sci. Rev.* **24**, 1063–1081 (2005).
21. DeLong, K. L., Quinn, T. M., Shen, C-C. & Lin, K. A snapshot of climate variability at Tahiti 9.5 ka using a fossil coral from IODP expedition 310, *Geochem. Geophys. Geosyst.* **11**, GC002758 (2010).
22. Asami, R. *et al*. Evidence for tropical South Pacific climate change during the Younger Dryas and the Bølling–Allerød from geochemical records of fossil Tahiti corals. *Earth Planet. Sci. Lett.* **288**, 96–107 (2009).
23. Stute, M. *et al*. Cooling of Tropical Brazil (5°C) During the Last Glacial Maximum. *Science* **269**, 379–383 (1995).
24. Guilderson, T. P., Fairbanks, R. G. & Rubenstone, J. L. Tropical Temperature Variations Since 20,000 Years Ago: Modulating Interhemispheric Climate Change. *Science* **263**, 663–665 (1994).
25. Gagan, M., Dunber, G. B. & Suzuki, A. The effect of skeletal mass accumulation in *Porites* on coral Sr/Ca and δ^18^O paleothermometry. *Paleoceanography* **27**, PA002215 (2012).
26. Durand, N. *et al*. Comparison of ^14^C and U-Th ages in corals from IODP #310 cores offshore Tahiti. *Radiocarbon* **55**, 1–26 (2013).
27. Hathorne, E. C., Felis, T., James, R. H. & Thomas, A. Laser ablation ICP-MS screening of corals for diagenetically affected areas applied to Tahiti corals from the last deglaciation. *Geochim. Cosmochim. Acta* **75**, 1490–1506 (2011).
28. Bard, E. *et al*. Deglacial sea-level record from Tahiti corals and the timing of global melt water discharge. *Nature* **382**, 241–244 (1996).
29. Cabioch, G. *et al*. Successive reef depositional events along the Marquesas foreslopes (French Polynesia) since 26 ka. *Mar. Geol.* **254**, 18–34 (2008).
30. Heindel, K., Wisshak, M. & Westphal, H. Microbioerosion in Tahitian reefs: a record of environmental change during the last deglacial sea-level rise (IODP 310). *Lethaia* **42**, 322–340 (2009).
